# Integrating Omics and CRISPR Technology for Identification and Verification of Genomic Safe Harbor Loci in the Chicken Genome

**DOI:** 10.1186/s12575-023-00210-5

**Published:** 2023-06-24

**Authors:** Nima Dehdilani, Lena Goshayeshi, Sara Yousefi Taemeh, Ahmad Reza Bahrami, Sylvie Rival Gervier, Bertrand Pain, Hesam Dehghani

**Affiliations:** 1 Stem Cell Biology and Regenerative Medicine Research Group, Research Institute of Biotechnology, Ferdowsi University of Mashhad, Azadi Square, Mashhad, Iran; 2 Division of Biotechnology, Faculty of Veterinary Medicine, Ferdowsi University of Mashhad, Mashhad, Iran; 3 Department of Biology, Faculty of Science, Ferdowsi University of Mashhad, Mashhad, Iran; 4 Industrial Biotechnology Research Group, Institute of Biotechnology, Ferdowsi University of Mashhad, Mashhad, Iran; 5 Stem Cell and Brain Research Institute, University of Lyon, Université Lyon 1, INSERM, INRAE, U1208, USC1361, 69500 Bron, France; 6 Department of Basic Sciences, Faculty of Veterinary Medicine, Ferdowsi University of Mashhad, Mashhad, Iran

**Keywords:** Genomic Safe Harbor Loci, Chicken Genome, CRISPR/Cas9 Technology, Stably-Expressed Transgene, Transgenesis, Weakened Promoter

## Abstract

**Background:**

One of the most prominent questions in the field of transgenesis is ‘Where in the genome to integrate a transgene?’. Escape from epigenetic silencing and promoter shutdown of the transgene needs reliable genomic safe harbor (GSH) loci. Advances in genome engineering technologies combined with multi-omics bioinformatics data have enabled rational evaluation of GSH loci in the host genome. Currently, no validated GSH loci have been evaluated in the chicken genome.

**Results:**

Here, we analyzed and experimentally examined two GSH loci in the genome of chicken cells. To this end, putative GSH loci including chicken HIPP-like (cHIPP; between DRG1 and EIF4ENIF1 genes) and chicken ROSA-like (cROSA; upstream of the THUMPD3 gene) were predicted using multi-omics bioinformatics data. Then, the durable expression of the transgene was validated by experimental characterization of continuously-cultured isogenous cell clones harboring DsRed2-ΔCMV-EGFP cassette in the predicted loci. The weakened form of the CMV promoter (ΔCMV) allowed the precise evaluation of GSH loci in a locus-dependent manner compared to the full-length CMV promoter.

**Conclusions:**

cHIPP and cROSA loci introduced in this study can be reliably exploited for consistent bio-manufacturing of recombinant proteins in the genetically-engineered chickens. Also, results showed that the genomic context dictates the expression of transgene controlled by ΔCMV in GSH loci.

**Supplementary Information:**

The online version contains supplementary material available at 10.1186/s12575-023-00210-5.

## Background

Epigenetic silencing and promoter shutdown are the main impediments ahead of reliable and consistent transgene expression over time [1, 2]. In this regard, both vector-dependent and host-dependent factors may affect the fate of transgene expression (reviewed in [2]). To avoid the effects of negative host-dependent factors on transgene expression, several research projects have tried to find the most appropriate target loci across the genome and to integrate the transgene therein [3–5]. In previous studies, intergenic [5], intronic [3], pseudo attP [6], mCreI [7], and pMEI [8] sites have been used as GSH loci to safely host and stably express the transgenes.

The CRISPR/Cas9 technology has revolutionized the genomic site-specific targeting of somatic and embryonic cells [9]. Site-specific integration into the predicted GSH loci and subsequent isolation of cells carrying monocopy transgene can lead to low clonal variations, and homogeneous as well as consistent gene expression [10, 11]. The in vitro evaluation of the long-term expression competency of a transgene integrated into a predicted GSH locus is very important and valuable before generating genetically engineered animals. This is to avoid the possible shutting down of the promoter or silencing of the transgene over time [5, 6].

In recent years, several attempts have been made to identify GSH regions [3, 7, 12–14] and to exploit them for efficient transgenesis in rodents [5, 15–18], mammals [19–22], and human [23], as well as manufacturing of recombinant protein in cell lines [10, 24, 25].

The following criteria may be considered for the evaluation of a candidate locus as a potential/putative GSH, mainly for biotechnological applications. i) GSH loci should be located in a stable chromosome with low rearrangements. The use of cells with a heavily-mutated genome, inappropriate genome integrations, and high chromosomal duplications should be avoided. In this regard, using primary cells or cells derived from non-cancerous tissues are preferable. ii) weak or cell-specific promoters should be used to evaluate the expression profile of a potential GSH locus, iii) the intronic regions should be preferably avoided since they are subject to transcription read-through events, iv) to minimize expression variations, isogenous cell clones harboring the transgene in the GSH locus should be used. The main feature of a GSH locus for biotechnological applications is its ability to support long-term and consistent expression in a population of cells.

Several GSH regions such as ROSA26 [26], HIPP [5], AAVS1 [27], and HPRT1 [28] have been used to host the transgene. Also, different heterologous and homologous promoters have been used to drive the transgene expression from these GSH regions [4, 29]. It has been demonstrated that the expression profile of a given transgene in a GSH locus would be promoter-, genome context-, copy number-, and orientation-dependent [30, 31]. Among these, the transgene expression can be strongly affected by the genomic context and the type of promoter over time [10]. In GSH studies, transgenes have been driven by strong/weak heterologous promoters [4], homologous promoters (especially cell/tissue-specific promoters) [4, 29], minimal promoters [29, 32], as well as promoter-less genomic regions [18, 19, 22, 33].

To evaluate GSH loci, exploiting strong heterologous or homologous promoters might be deceptive/misleading and lead to unpredictable results. Insertion of strong promoters in the genome would not necessarily lead to the ubiquitous expression of the reporter [10, 29]. In most previous studies, the identification of GSH loci has been conducted by a strong promoter. However, by using weak or tissue-specific promoters, the prediction of the potential GSH regions would be more realistic [19, 29, 34, 35]. In addition, the investigation of expression profiles of the integrated transgene in a potential GSH locus should be carried out in parallel with the integration of the same transgene in a non-GSH locus [6]. To our knowledge, there is no report regarding the evaluation of GSH loci using a transgene driven by a weak promoter that is simultaneously integrated both in GSH and non-GSH loci.

There are different strategies that could be used for screening the genomic loci to predict the safe loci for transgene knock-in; i) the traditional gene trapping method which relies on random integration of reporter construct followed by isolating the cells with higher expression [36, 37] or whole genome sequencing to predict the reporter integration sites that are supporting the high expression of the reporter [38], ii) random integration of the transgene by homologous recombination followed by in-vivo imaging to identify the permissive loci for the generation of stably-expressed reporter animal [29], iii) Mining of the genomic [3, 7, 14, 39, 40], the epigenomic [13, 41], and the transcriptomic [41] data based on registered data in bioinformatic databases, iv) Comparative genomics for screening and detecting the similarity between GSH in one species and other species based on homologous sequences [12, 18, 21, 22].

Here, using multi-omics bioinformatics such as comparative genomics approach, transcriptomics data, and Hi-C data, two novel GSH loci including cROSA and cHIPP were predicted in the chicken genome. The similarity in the sequences or neighboring gene arrangement with ROSA26 [12, 18, 21, 22] and H11 [19, 23, 42, 43] loci were used to predict these potential GSH regions in the chicken genome. These two loci are located in micro-chromosomes 12 and 15 which are proven as hyperacetylated and highly-transcribed chromosomes and have high stability against the rearrangement and insertion of repetitive elements [44].

It has been demonstrated that the transgene expression from intergenic regions is highly preferable to that from intragenic regions [3, 5]. Although intragenic regions such as ROSA26, CCR5, and AAVS1 were successfully implemented for safe and durable transgene expression in different cell types, there are some constraints. For example, the insertion of transgenes in the AAVS1 region directly affects the growth rate of engineered cells following transgene silencing [45]. Also, endogenous promotor located in the ROSA26 locus leads to the ubiquitous expression of the transgene and may influence transgene expression driven by exogenous promoters. Mosaic expression of the transgene in multiple organs was reported when the CAG promoter was used in the ROSA26 locus [46, 47]. Silencing of the transgene can occur in the CCR5 locus due to the 0.9 kb sequence that is susceptible to methylation [4]. Thus, it seems that the use of intergenic loci to host the transgene for biotechnological applications is advantageous over the intragenic loci. Higher rate of recombination, targeting efficiency, and level of transgene expression in vivo as well as stable transgene expression without silencing are the features of an intergenic locus such as H11 [5].

We used CRISPR/Cas9 technology to integrate the EGFP transgene under the control of strong CMV and weakened ΔCMV promoter in the identified GSH loci as well as in the non-GSH ovalbumin locus (hereafter called cOVA) in the chicken fibroblast cell lines (DF1 cell lines). In contrast to our expectation, the transgene expression under the control of a CMV promoter in a non-GSH locus outperformed the one integrated into the GSH locus. This result suggested that the expression of a transgene controlled by a strong promoter may act independently from its chromosomal position effects. Replacing the CMV promoter with ΔCMV altered the expression level in favor of GSH loci. To fully decipher the behavior of GSH loci, isogenous cell clones harboring ΔCMV promoter-driven EGFP were isolated. The results revealed that mono-allelic and mono-copy expression levels of EGFP controlled by ΔCMV promoter in GSH loci were significantly higher than that in non-GSH locus. Also, we found that DF1 cell lines that have integrated the transgene cassette in the GSH locus can express EGFP for more than 6 months. For applications in biotechnology, consistent and homogenous level of expression of the transgene is preferred [30].

## Materials and Methods

### Bioinformatic Identification of GSH Loci

**Fig. 1:**
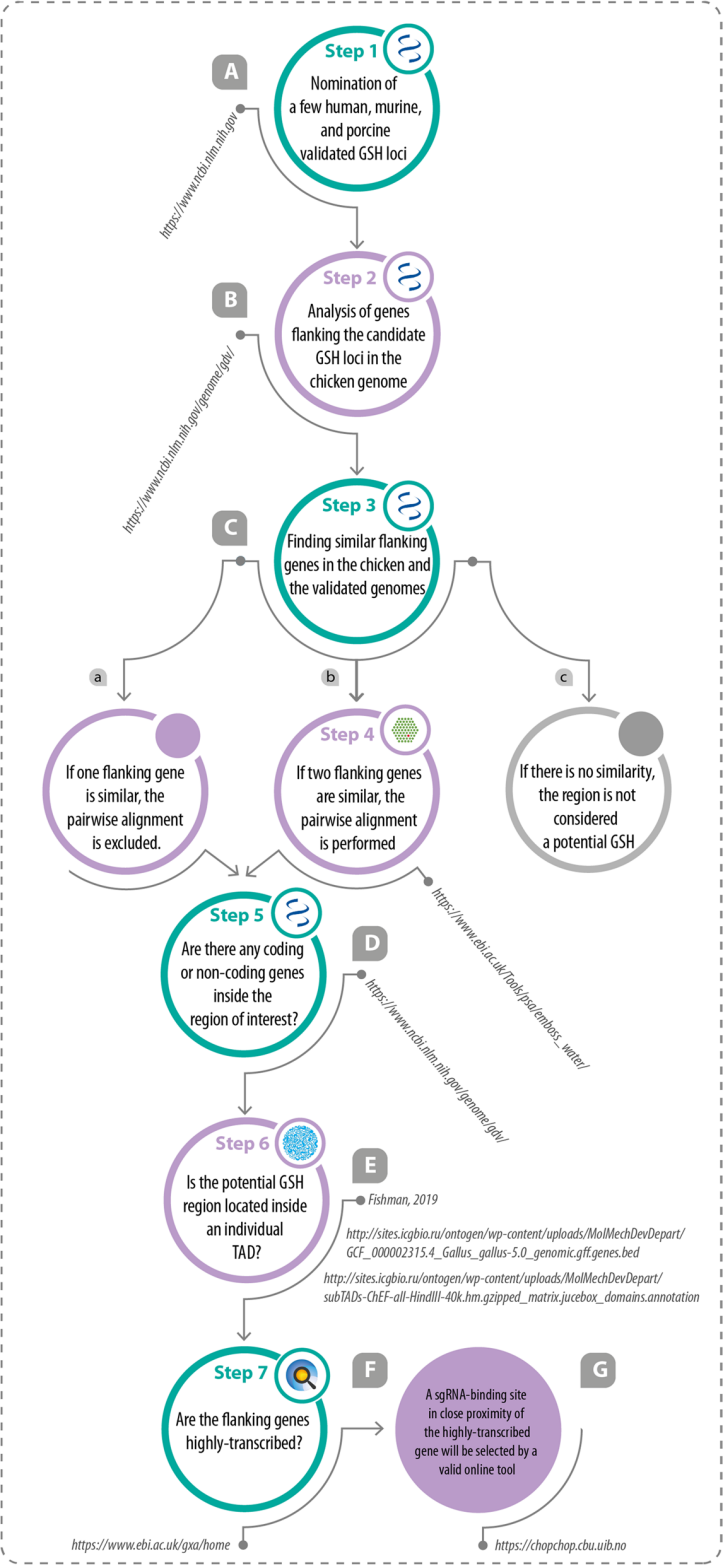
A schematic pipeline for the prediction of genomic safe harbor loci in the chicken genome. Seven steps were followed to predict the chicken genomic safe harbor loci. Step 1) A few validated GSH loci in the human, murine, and porcine genomes were nominated (**A**). Step 2) Flanking genes surrounding the validated GSH loci were analyzed in the chicken genome using the genome data viewer of NCBI (**B**). Step 3) Gene arrangement around the validated GSH loci was compared to the potential GSH locus in the chicken genome (**C**). Step 4) If one flanking gene was similar, the pairwise alignment was excluded and the process was followed from step 5 (**C**-**a**). If two flanking genes were similar, pairwise alignment was performed (**C**-**b**). And if there was no similarity, the region was not considered a potential GSH (**C**–**c**). Step 5) The presence of possible annotated coding or non-coding genes in the predicted GSH locus was evaluated by GDV (Gallus gallus genome assembly bGalGal1.GRCg7b/w) and the UCSC Genome Browser (**D**). Step 6) The coordinates of predicted GSH loci were evaluated by Hi-C data to ensure that the insertion site was contained within an individual TAD (**E**). Step 7) The expression levels of the genes flanking the potential GSH locus were accessed from Gene Expression Atlas to determine whether these genes are highly-transcribed (**F**). A sgRNA-binding site in close proximity of the highly-transcribed gene will be selected by a valid online tool (**G**)

To identify and characterize the potential GSH loci in the chicken (*Gallus gallus domesticus*) genome, we applied a stepwise multi-omics bioinformatics strategy (Fig. 1). Validated GSH loci (HIPP/H11 and Gt ROSA 26Sor/ROSA26 loci in human, murine, and porcine genomes) were analyzed using the NCBI’s genome data viewer (https://www.ncbi.nlm.nih.gov/genome/gdv/) (Fig. 1A). Then, flanking genes of these GSH loci were determined (Fig. 1B). If the arrangement of two flanking genes in the chicken genome was similar to the orthologous genomes, these regions were aligned together (Fig. 1C-b). To this end, pairwise alignment of the validated and candidate intergenic region was conducted by EMBOSS WATER algorithm (https://www.ebi.ac.uk/Tools/psa/emboss_water/). The alignment was excluded if only one flanking gene around the validated GSH locus was similar to that in the chicken genome (Fig. 1C-a). If there was no similarity, the region was not considered as a potential GSH (Fig. 1C-c). The presence of possible coding or non-coding genes in the potential GSH locus was evaluated by NCBI’s genome data viewer or UCSC genome browser (https://genome.ucsc.edu) (Fig. 1D). Chicken Hi-C data was used to study whether the potential GSH is contained within an individual TADs (Fig. 1E and Additional file 1). Hi-C data was visualized by Juicebox software (version 1.11.08) which is available at the following link: https://github.com/aidenlab/Juicebox/wiki/Download. Coordinates of defined TAD boundaries are shown in Additional file 2 and coordinates of the annotated genes are shown in Additional file 3. The coordinate system of the map corresponds to the genome version *GalGal5*. After visualizing the data by Juicebox, the chromosome containing the genes of interest (DRG1, EIF4ENIF1, THUMPD3, flanking the potential chicken GSH locus, and OVAL and OVALY flanking the a non-GSH locus) was selected and visualized at 5 kb resolution.

Chicken RNAseq data in the Gene Expression Atlas (https://www.ebi.ac.uk/gxa/home) were used to determine the transcript level of the flanking genes (Fig. 1F) in embryonic and adult stages. In the Gene Expression Atlas, the expression heatmap included four defined cut-off levels: below (0.5 TPM; transcript per million), low (0.5 to 10 TPM), medium (11 to 1000 TPM), and high (more than 1000 TPM).

### Preparation of Targeting Vectors

In order to construct cROSA, cHIPP, and cOVA targeting vectors, 5’ and 3’ homology arm (HA) sequences spanning the sgRNA target sites were amplified from the genomic DNA (gDNA) of DF1 cell lines (chicken embryonic fibroblast cell lines) (Additional file 4) by the specific primer sets (Additional file 5; primers P1 to P4 for amplification of cROSA left HA, primers P5 to P8 for amplification of cROSA right HA, primers P9 and P10 for amplification of cHIPP left HA, primers P11 and P12 for amplification of cHIPP right HA, primers P13 to P16 for amplification of cOVA left HA, and primers P17 to P20 for amplification of cOVA right HA). To generate cHIPP targeting vector (VH), cROSA targeting vector (VR), and cOVA targeting vector (VO), the 5’ and 3’ HA sequences related to each locus were sub-cloned into the PvuI/XhoI and NheI/XcmI sites of a vector containing DsRed2-PolyAsignal-CMV-EGFP-IRES-PAC^r^-PolyAsignal (hereafter called DsRed2-CMV-EGFP), respectively (Fig. 3A, B-a, C-a, D-a). To generate targeting vectors with a weakend form of CMV promoter (hereafter called ΔCMV promoter), the whole distal part of CMV and a part of proximal CMV from BglII to SnaBI restriction sites was removed (Fig. 4B). These vectors containing DsRed2-PolyAsignal-ΔCMV-EGFP-IRES-PAC^r^-PolyAsignal cassette (hereafter called DsRed2-ΔCMV-EGFP) were named ΔVH, ΔVR, and ΔVO (Fig. 4C-a, D-a, E-a). We used the promoterless DsRed2 reporter in the final targeting vectors to monitor any upregulatory effects of the GSH locus. The site-specific sgRNA oligonucleotides (for cROSA: 5’-tccgggtcggtttggcccct-3’, for cHIPP: 5’-gcctgtactttgttagtgac-3’, and for cOVA: 5’-gctctagccatggtatacct-3’) were designed by CHOPCHOP (https://chopchop.cbu.uib.no) online software (Fig. 1G; Additional file 6) and were cloned into the BbsI sites downstream of the U6 promoter in a Cas9/gRNA vector containing CBA-Cas9-T2A-PAC^r^. All vectors contained puromycin-resistance gene indicated by PAC^r^.

### Cell Culture, Transfection, and Generation of Heterogenous Cell Pools and Isogenous Cell Clones

DF1 cells were grown in Dulbecco’s Modified Eagle Medium/Nutrient Mixture F-12 (DMEM/F12) (Gibco, USA) supplemented with 10% fetal bovine serum (Gibco, USA), and penicillin (10,000 I.U./mL)/streptomycin (10,000 μg/mL) (Thermo Fisher Scientific, USA). To achieve stable heterogenous EGFP-expressing cell pools, VH, VR, VO, ΔVH, ΔVR, and ΔVO targeting vectors (1ug/ul from each) along with the corresponding specific Cas9/gRNA vectors (1 ug/ul from each) were co-transfected into the DF1 cells (2 × 10^5^ cells per 12 well-plate) using Lipofectamine 3000 (Invitrogen, USA) in six different experimental groups (in three biological replicates). These groups included cells with CMV-driven EGFP and ΔCMV-driven EGFP in three loci of cHIPP, cROSA, and cOVA (Additional file 7B, C). Transfection was followed by a 1-week selection with 1 μg/mL of puromycin (Sigma-Aldrich, USA) and another week for recovery (Additional file 7A). At the end of month two (MTH2), the heterogeneous cell pools carrying DsRed2-ΔCMV-EGFP in each of the three loci were subjected to limiting dilution to achieve the isogenous cell clones that stably expressed EGFP (Additional file 7C). At the end of months four (MTH4) and six (MTH6), each group of long-term cultured isogenous cell clones was evaluated for EGFP and DsRed2 expression (Additional file 7D, E). We reasoned that any possible DsRed2 expression without any promoter may indicate the influence of the genomic context in the relevant GSH locus. To make control groups, the sgRNA-free Cas9 (-gRNA) vector (1 ug/ul) was co-transfected with each of the VH, VR, VO, ΔVH, ΔVR, and ΔVO (1ug/ul from each) targeting vectors. The expression of EGFP and DsRed2 was assessed in heterogenous cell pools at the end of MTH2 (Additional file 7B and 7C), and in isogenous cell clones isolated from the long-term culture (> 2 months) of heterogenous cell pools at the end of MTH4 (Additional file 7D) and MTH6 (Additional file 7E).

### Limiting Dilution to Isolate Isogenous Cell Clones

For each experimental group, a total number of 100 heterogeneous cells carrying DsRed2-ΔCMV-EGFP were counted, resuspended in 1000 µl of complete medium, and dispensed in 96 well plates (10 µl/well) containing 90ul medium per well. Isogenous cell clones appeared within 2 weeks of culture (Additional file 8A). Then, individual isogenous cell clones were picked up and expanded in 24 well-plates 1 month more. These cells were continuously maintained for more than 6 months in culture.

### Verification of Bi- or Mono-Allelic Integration of the dsRed2-ΔCMV-EGFP Cassete

Six isogenous cell clones were evaluated for bi- or mono-allelic integration of the DsRed2-ΔCMV-EGFP cassete for each gene locus (Additional file 8B). To this end, three different PCR reactions were performed to verify the mono-allelic integration events; using P1/GS2 and VS2/GS2 primer pairs for the cROSA locus, GS3/GS7 and VS2/GS4 primer pairs for the cHIPP locus, and P13/GS6 and VS2/GS6 primer pairs for the cOVA locus (Additional file 5).

### Verification of On-Target Integration

Correctly-knocked-in heterogenous cell pools and isogenous cell clones were confirmed by 5’/3’ junction PCR. GS1-VS1, GS3-VS1, and GS5-VS1 primer pairs were used to confirm on-target integration in the 5’ junction of cROSA, cHIPP, and cOVA loci, respectively. In addition, GS2-VS2, GS4-VS2, and GS6-VS2 primer pairs were used to validate on-target integration in the 3’ junction of these loci, respectively (Figs. 3 and 4, Additional file 5, Additional files 8C, 9). Amplified fragments from the 5’ and 3’ sides of each locus by the above-mentioned primer pairs were confirmed by restriction enzyme digestion and Sanger sequencing (Additional files 8D, 9, 10).

### Flowcytometry

For the flow cytometric analysis, heterogenous cell pools (containing either CMV or ΔCMV) from each experimental group (cROSA, cHIPP, and cOVA) were analyzed using FACS Calibur (BD Biosciences, USA) in three biological replicates at the end of MTH2. To this end, 3 $\times$ 10^5^ cells were counted, washed twice with cold D-PBS (DENAzist Asia, Iran), and resuspended in cold D-PBS. Then, the percentage of EGFP- and DsRed2-positive cells were acquired in green and red channels, respectively. The mean fluorescence intensity (MFI) index was calculated by FlowJo software (version 7.0) for all experimental groups. A comparison of the MFI index was performed among the experimental groups where the cOVA group was used as a control group for both cROSA and cHIPP groups. Similarly, isogenous cell clones were analyzed at the end of MTH4 and MTH6 to determine the percentage of EGFP- and DsRed2-positive cells as well as the MFI index.

### Image Analysis Using ImageJ and Gnuastro Softwares

The images captured from each group of heterogeneous cell pools (containing either CMV or ΔCMV promoter) were analyzed by ImageJ Fiji software (version 1.52p) to calculate the integrity density index. For this purpose, cells were imaged with identical parameters (for images from CMV groups: 10X magnification, 100 µs exposure, 1X analog gain, and for images from ΔCMV groups: 20X magnification, 1 s exposure, 2.2X analog gain). Also, isogenous cell clones containing ΔCMV promoter were analyzed to calculate the integrity density index. These cells were also imaged with identical parameters (20X magnification, 1 s exposure, 1X analog gain). The integrity density index was further analyzed by GNUastro Linux-based software (version 0.18) for images taken from isogenous cell lines containing ΔCMV promoter (Additional file 11).

### qPCR Quantification of EGFP Transcripts

Total RNA was extracted from cells in each biological replicates using the Column RNA Isolation Kit (DENAzist Asia, Iran). A total of 2000 ng RNA was reverse transcribed using RevertAid First Strand cDNA Synthesis Kit (Thermo Fisher Scientific, USA). qPCR was conducted with 200 ng cDNA in a 20 µl reaction using the RealQ Plus 2 × Master Mix Green (Ampliqon, Denmark) and Rotor-Gene TM6000 Real-Time PCR Analyzer (Qiagen, Germany) in three technical replicates. Primer-pair for EGFP (P21/P22) and chicken ACTB (P23/P24) was used to amplify EGFP and ACTB transcripts, respectively (Additional file 5). All reactions were subjected to 35 cycles of initial denaturation for 15 m at 94 °C, 30 s denaturation at 94 °C, 30 s annealing at 68 °C for EGFP primer-pair and 55°C for chicken ACTB primer-pair, and 30 s extension at 72 °C. The melting curve was acquired between 45 °C and 95 °C. The size of the EGFP and chicken ACTB amplicons were 174 bp and 118 bp, respectively. One to five dilution series of EGFP cDNA was prepared and used to generate a standard curve using the SYBR Green qPCR mastermix (Ampliqon, Denmark) by amplifying EGFP. To this end, 2 µl of the cDNA from each dilution was added to 18 µl SYBR Green qPCR Mastermix in three technical replicates. The logarithm of the initial copy number of transcripts was plotted along the x-axis, and their respective C_T_ values were plotted along the y-axis. Based on the equation for the linear regression, the following equation was used to determine the copy number of the EGFP mRNA in the heterogenous cell pools and isogenous cell clones based on the standard curve equation formula.

$$\mathrm{Copy}\;\mathrm{number}\;=10\hat{}\left(\frac{n-b}m\right),$$

where n = C_T_ for each sample, b = 53.7092, m = -3.6916

Chicken ACTB was used as a house-keeping mRNA for normalization of the data. To this end, the average C_T_ of ACTB in the cHIPP group was devided individually to the average C_T_ of ROSA and OVA groups. Then, C_T_ of EGFP of ROSA and OVA groups was devided by the calculated ratios. The copy number of EGFP transcripts was calculated using the standard curve equation formula.

### Western Blotting Analysis of EGFP Expression

Total protein was extracted from isogenous cell clones carrying DsRed2- ΔCMV-EGFP using RIPA lysis buffer (Cytomatin gene, Iran). Briefly, 50 µl RIPA lysis buffer was poured on the cells cultured in a 0.35 cm^2^ cell culture dish after removing the culture medium, followed by 30 min incubation in a minus 30 freezer. Then, cells were scraped and centrifuged in 4 °C with a speed of 13,000 rpm for 5 min. Total protein was collected from the supernatant and stored at a minus 80 freezer. The BCA protein assay was conducted to determine the concentration of total protein. Each protein sample (30 μg) was subjected to 10% SDS-PAGE, transferred onto 0.45 µm nitrocellulose membrane (Biorad, USA) according to the standard protocol, followed by blocking (overnight at 4 °C) with 5% w/v skim milk prepared in D-PBS. The membrane was incubated with rabbit monoclonal antibody (Sino Biological Inc., China) against EGFP (1:2000) for 3 h at room temperature, and with HRP-conjugated goat anti-rabbit secondary antibody (1/18000) (Abcam, UK) for 1 h at room temperature. As an internal control, chicken β-actin protein was used to normalize the results. To this end, stripping of the same membrane was performed using a standard protocol and blocking was conducted with 5% skim milk overnight at 4 °C. Then, the membrane was subjected to a mouse monoclonal anti-β-actin antibody (1/20000) (Sigma Aldrich, USA) for 1 h at room temperature. Throughout the experiments, each membrane was washed three times (15 min per wash) using TBST solution. Finally, in order to detect the HRP signals, membranes were subjected to chemiluminescence detection with the Chemiluminescence Kit (Parstous, Iran), and the Chemiluminescence Detector system (G: BOX Chemi XT Analyser, SYNGENE, Eur.). The ImageJ software was used for densitometric analysis of the EGFP (27 kilodalton) and chicken β-actin (42 kilodalton) protein bands. Mean relative intensity was calculated and Mann–Whitney test was used to compare the relative intensity among the analyzed groups (*p* < 0.05).

### Determination of the Copy Number of Integrated EGFP Transgene in Isogenous Cell Clones

Genomic DNA of non-transfected DF1 cells was isolated using the Animal Tissue DNA Isolation Kit (DENAzist Asia, Iran). Extraction of EGFP-containing plasmid was performed using the Plasmid Isolation Kit (DENAzist Asia, Iran). The copy number of extracted gDNA and plasmid were calculated by the following formula:

$$\mathrm{Number}\;\mathrm{of}\;\mathrm{copies}\;\mathrm{of}\;\mathrm{DNA}\;\mathrm{template}\;\mathrm{per}\;\mu\mathrm l=\frac{\mathrm{DNA}\;\mathrm{concentration}\;\left({\displaystyle\frac{\mathrm{ng}}{\mu\mathrm l}}\right)\times\;\mathrm{Avogadro}'\mathrm s\;\mathrm{number}\;\left[6.022\;\times10^{23}\right]}{\mathrm{length}\;\mathrm{of}\;\mathrm{template}\;\left(\mathrm{bp}\right)\times\mathrm{conversion}\;\mathrm{factor}\;\mathrm{to}\;\mathrm{ng}\;\left[10^9\right]\;\times\;\mathrm{average}\;\mathrm{weight}\;\mathrm{of}\;\mathrm a\;\mathrm{base}\;\mathrm{pair}\;\left(\mathrm{Da}\right)\;\left[650\right]}$$

The haploid size of chicken genome (1.05333 Mb according to assembly GCA_016699485.1 bGalGal1.mat.broiler.GRCg7b) was used to calculate the copy number of extracted gDNA. Then, the volume containing equal to one-hundred copies of the diploid genome was mixed with a volume containing equal to one-hundred copies of the plasmid (i.e., ratio 1:1). Then, a 1:5 dilution series of the mix was prepared and was used to generate a standard curve using the SYBR Green qPCR mastermix (Ampliqun, Denmark) by amplifying EGFP. To this end, 2 µl of the mix from each dilution was added to 18 µl SYBR Green qPCR Mastermix in three technical replicates. The logarithm of the initial copy number of genomes (containing one copy of EGFP per genome) was plotted along the x-axis, and their respective C_T_ values were plotted along the y-axis (Additional file 8E). Based on the equation for the linear regression, the following equation was used to determine the copy of the EGFP transgene in the genome of isogenous cell clones with similar mass (extracted genomic DNA in ng).

$$\mathrm{Copy}\;\mathrm{number}\;=10\hat{}\left(\frac{n-b}m\right),$$

where n = C_T_ for each sample, b = 21.707, m = -3.611

Genomic DNA of isogenous cell clones and heterogenous cell pools was isolated using the Animal Tissue DNA Isolation Kit (DENAzist Asia, Iran). 10 ng from each gDNA (coresponding to 8662.28 genome copies) was added to 18 µl SYBR Green qPCR Mastermix in three technical replicates. Using the above-mentioned equation, EGFP copy numbers were calculated in each cell clone and cell pool.

## Results

### Bioinformatic Analysis for Predication of Genome Safe Harbor Loci in the Chicken Genome

**Fig. 2:**
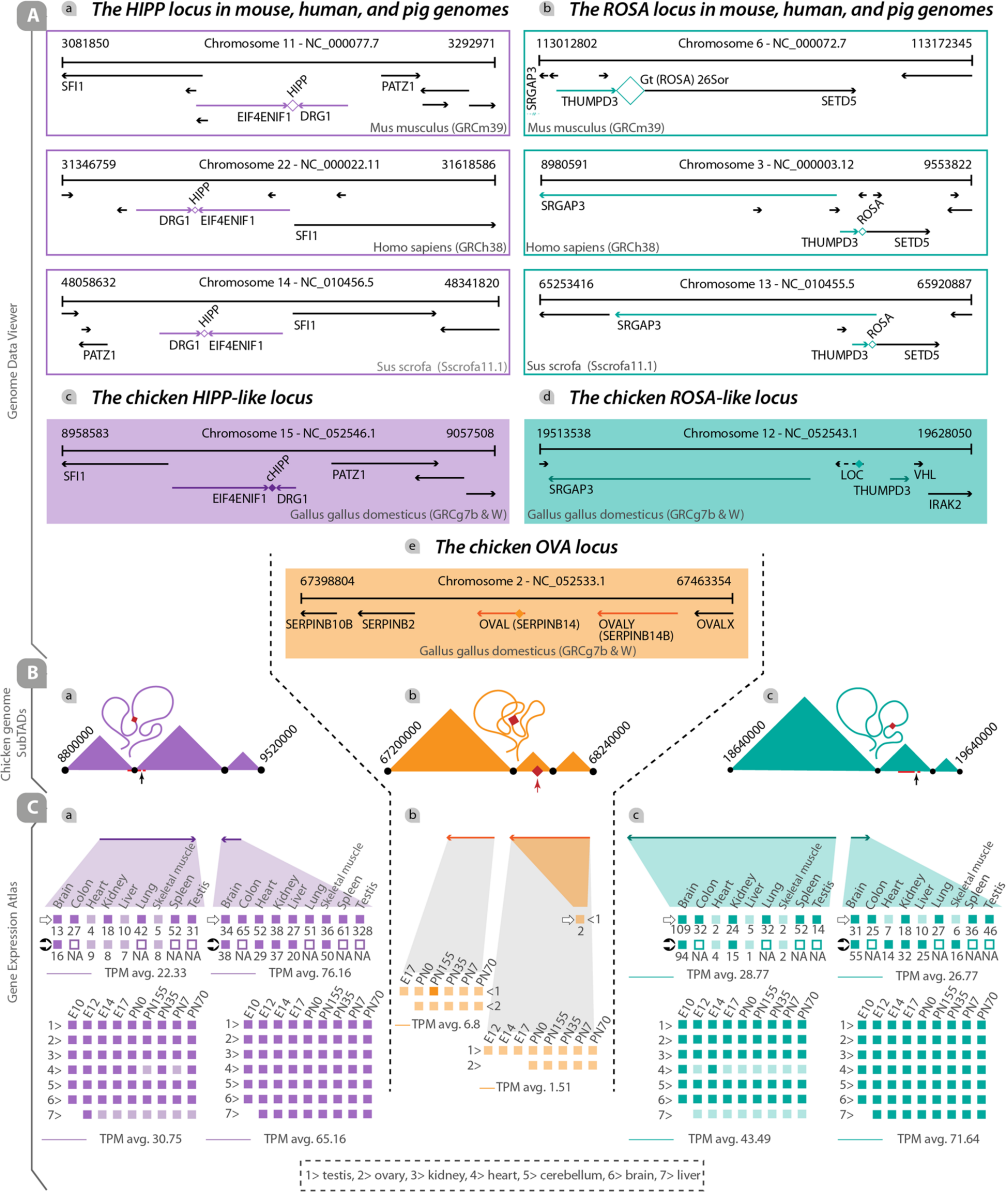
Bioinformatic analysis for predication of genome safe harbor loci in the chicken genome. **A-a** The schematic presentation of the validated HIPP locus including its flanking genes in the mouse, human, and pig genomes. **A**-**b **The schematic presentation of the validated ROSA locus including its flanking genes in the mouse, human, and pig genomes. **A**-**c**, **A**-**d **The schematic presentation of the potential cHIPP and cROSA loci in the chicken genome. Flanking genes around the validated HIPP locus (i.e., DRG1/ EIF4ENIF1) have been exactly the same as the genes found around the predicted cHIPP locus in the chicken genome, but the genes surrounding the validated ROSA locus (i.e., THUMPD3/SETD5/SRGAP3) have been relatively the same as the genes seen around the predicted cROSA locus. **A**-**e **The schematic presentation of the non-GSH cOVA locus in the chicken genome. **B** The coordinates of DRG1/EIF4ENIF1, THUMPD3/SRGAP3, and OVAL genes relative to the location of TADs, extracted from the chicken Hi-C data, and visualized by JUICEBOX online software (adopted from ref [48]). **C** The expression levels of the flanking genes in several tissues and developmental stages, adopted from the Gene Expression Atlas. ⇨: (adopted from ref [49]). ➲: (adopted from ref [50]). TPM avg.: transcripts per million averages. E: embryonic day. PN: post natal day

A multiomics bioinformatics pipeline was used to predict potential GSH loci in the chicken genome (*Gallus gallus domesticus*) (Fig. 1). Genome data viewer (Fig. 2A), Hi-C data (Fig. 2B and Additional file 1), and RNA-seq data (Fig. 2C) were exploited to predict the potential GSH loci in the chicken genome. Based on two well-known GSH loci, HIPP (so-called H11) and Gt (ROSA) 26Sor (so-called ROSA26) which are validated GSH loci in several organisms including mice, humans, and pigs, we first evaluated the genes surrounding these intergenic loci. HIPP and ROSA26 intergenic loci are surrounded by EIF4ENIF1/DRG1 and THUMPD3/SETD5 genes, respectively in mouse, human, and pig.

Our survey using the genome data viewer of NCBI revealed that the EIF4ENIF1/DRG1 genomic arrangement in the chicken genome (Fig. 2A-c) was similar to those in the indicated organisms (Fig. 2A-a). Pairwise alignment (EMBOSS Water algorithm) was used to find the percentage of identity and similarity of the intergenic sequences between the EIF4ENIF1/DRG1 genes in the chicken genome with the same intergenic sequence in the mouse, human, and pig genomes. Results showed that this locus in chicken had 35.9%, 44.6%, and 40.9% similarity with the corresponding region in the mouse, human, and pig genomes, respectively (data not shown).

Contrary to what we found in the mouse, human, and pig genomes (Fig. 2A-b), the SETD5 gene was not adjacent to the THUMPD3 in the chicken genome (Fig. 2A-d). So, we were unable to use the intergenic sequence located between THUMPD3/SETD5 genes as a potential intergenic region. We noticed that the arrangement and order of SRGAP3/THUMPD3 genes in the genome of the mouse, human, and pig (Fig. 2A-b) was exactly similar to those in the chicken genome (Fig. 2A-d). Thus, two regions were chosen as GSH candidates in the chicken genome without considering the similarity of sequences with other organisms; i) the intergenic region (14327 bp) between SETD5 and PLNXB3 genes (data not shown), and ii) the intergenic region (20105 bp) between THUMPD3 and SRGAP3 genes (Fig. 2A-d). The former contains two “LOC” genes (unpublished/undetermined genes; data not shown) and the latter contains one “LOC” gene. It has been demonstrated that some unidentified coding or non-coding genes may reside in the intergenic regions and affect the expression of the integrated transgene [51]. Thus, we chose the upstream region of the THUMPD3 gene which is a wide intergenic region (Fig. 2A-d) and compared it with the upstream region of the SETD5 (data not shown). This is a gene-poor intergenic region compared with the upstream intergenic region of the SETD5 which is a gene-rich region. Consciously, we decided to integrate the transgene into the unpublished/undetermined gene named “LOC121106669” (the targeted site is located 7742 bp upstream of the THUMPD3 gene inside the “LOC121106669” gene).

**Fig. 3:**
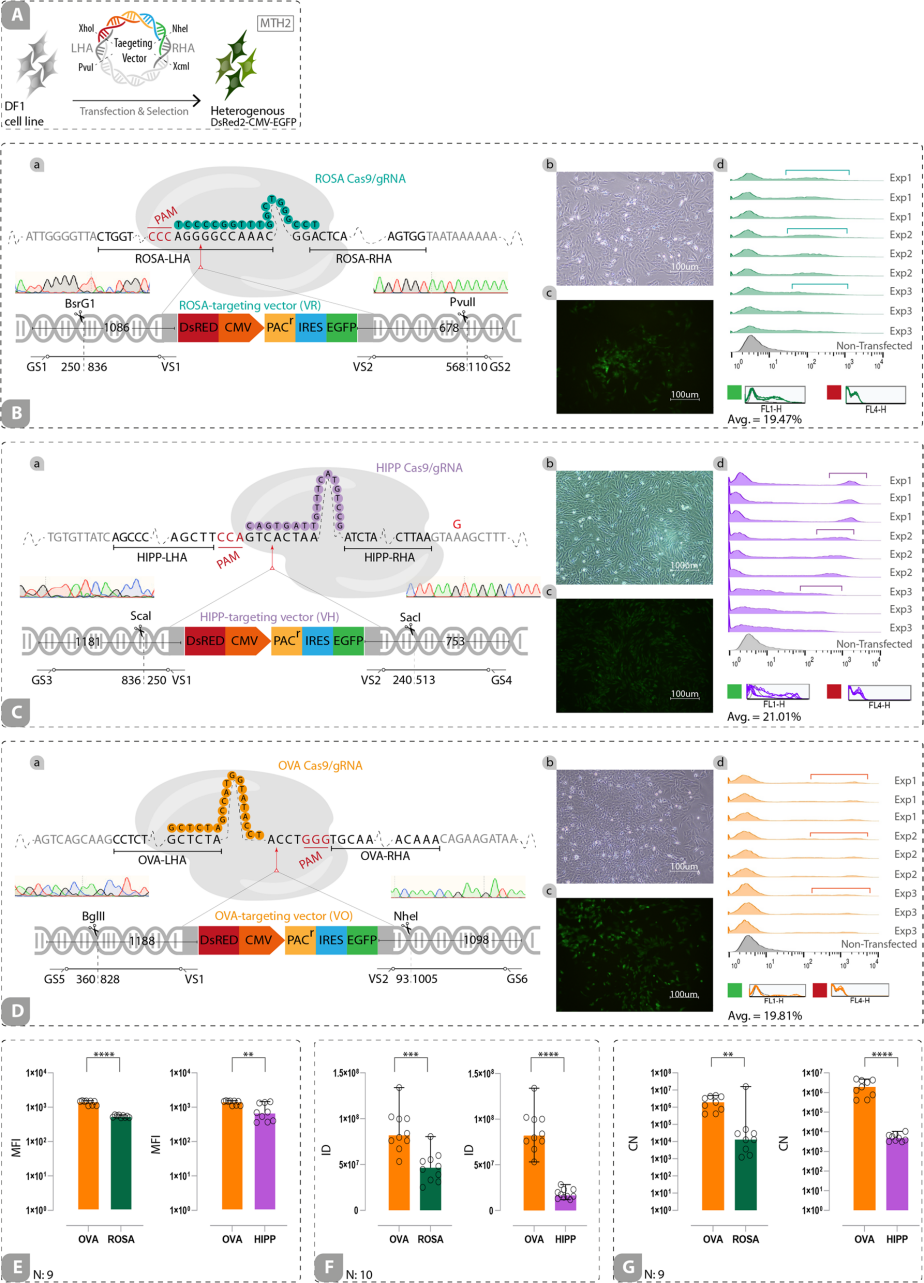
Transgene Expression from the strong heterologous promoter is not entirely locus-dependent. CRISPR-mediated integration of CMV-driven EGFP and promoter-less DsRed2 in the predicted GSH loci and non-GSH locus was performed in DF1 cell lines. **A** Schematic depiction of CMV-EGFP-expressing heterogeneous cell pools at the end of MTH2. (B-a, C-a, D-a) CRISPR-mediated integration of DsRed2-CMV-EGFP in cROSA, cHIPP, and cOVA loci. (B-b, B-c, C-b, C–c, D-b, D-c) Light and fluorescence microscope images of the cells expressing EGFP heterogeneously driven by the CMV promoter (Scale bar: 100μm). (B-d, C-d, D-d) Flow cytometry results from EGFP-expressing cROSA, cHIPP, and cOVA cells (each in triplicate). Non-transfected cells were used as the negative control. No expression signal was detected in the red channel. **E** Mean fluorescent intensity (MFI) index of the cOVA group was higher than that in the cHIPP and cROSA groups. **F** The integrated density (ID) index of the cOVA group was higher than that in the cHIPP and cROSA groups. **G** The copy number (CN) of EGFP transcripts in the cOVA group was higher than that in the cHIPP and cROSA groups. **: *p* < 0.05, ***: *p* < 0.005, and ****: *p* < 0.0001 are statistically significant. Avg.: The average expression of EGFP. Exp: Experiment. N: Number

Evaluating the chicken genome TADs revealed that both cHIPP and cROSA loci were located inside the individual TADs (Fig. 2B-a, B-c, and Additional file 12a, b). Also, the cOVA locus resides inside an individual TAD (Fig. 3B-b and Additional file 12c). On the other hand, chicken RNA-seq data were adopted to evaluate the expression levels (transcript per million; TPM) of the genes flanking the intergenic locus of interest (Fig. 2C-a, C–c). Since the expression levels of DRG1 and THUMPD3 outweighed those of EIF4ENIF1 and SRGAP3 genes, respectively, we decided to target the cHIPP and cROSA loci near these genes. TPM average for the DRG1 gene was 76.16 and 65.16 in tissues and developmental stages, respectively. TPM average for the EIF4ENIF1 gene was much less (22.33 in several tissues and 30.75 for developmental stages) (Fig. 2C-a). TPM average for the THUMPD3 gene was 26.67 and 71.64, in tissues and developmental stages, respectively. This average for the SRGAP3 gene was 28.77 and 43.49 in tissues and developmental stages, respectively (Fig. 2C-c). For the OVA gene, the TPM average was 6.8 in developmental stages, but no expression was reported in tissues. Low TPM is only observed in testis for OVALY, while it is below the cutoff in other tissues. Also, TPM in developmental stages is low for the OVALY gene (Fig. 2C-b). Hence, cROSA and cHIPP were nominated as the potential GSH loci, and cOVA was used as a non-GSH locus. Also, we evaluated OVA gene expression in DF1 cells and found that this locus is not transcriptionally active in DF1 cells (data not shown).

### Transgene Expression from the Strong Heterologous Promoter is not Entirely Locus-Dependent

In the first preliminary study, to evaluate the predicted GSH loci, a construct containing DsRed2-CMV-EGFP-IRES-PAC^r^ was inserted into two predicted cROSA and cHIPP loci as well as the non-GSH cOVA locus of chicken DF1 cells. Heterogenous cell pools (in triplicate for each locus) were generated by 1-week puromycin selection, followed by two-month culture without selection (Additional file 7A and Fig. 3A, B-a, C-a, D-a).

CRISPR-mediated knock-ins of construct harboring strong heterologous promoter in the designated loci were verified by 5’/3’ junction PCR (Additional file 9A-a, A-d, B-a, B-d, C-a, C-d), restriction enzyme digestion of the amplicons (Additional file 9A-c, B-c, C–c), and Sanger sequencing (Additional file 10A-a, B-a, C-a). When cells were transfected with a gRNA-free Cas9 vector (-gRNA), no integrations were observed, judged by 5’/3’ junction PCR (Additional file 9A-a, A-d, B-a, B-d, C-a, C-d) and a lack of EGFP flourscence (data not shown).

At the end of MTH2, correctly-knocked-in heterogenous cell pools for each locus/replicate were evaluated by flow cytometry to estimate the percentage of EGFP-positive cells. Results showed that 19.47%, 21.01%, and 19.81% of cells targeted in cROSA, cHIPP, and cOVA loci, respectively were EGFP-positive (Fig. 3B-d, C-d, D-d). The EGFP expression was highly variable in heterogenous cell pools, indicated by wide histograms (Fig. 3B-d, C-d, D-d). Also, no expression of the promoter-less DsRed2 was detected in any of the three loci (red square), judged by flow cytometry (Fig. 3B-d, C-d, D-d). MFI index showed that the expression of CMV-EGFP inserted in the cOVA locus was significantly higher than that for the CMV-EGFP knocked-in reporter in the cROSA and cHIPP loci (*p* < 0.0001 and < 0.05, respectively; Fig. 3E). Analyzed images captured from each locus (Fig. 3B-b, B-c, C-b, C–c, D-b, D-c, and Additional file 13) showed that the ID index of CMV-EGFP inserted in the cOVA locus was significantly higher than that for the CMV-EGFP knocked-in reporter in the cROSA and cHIPP loci (*p* < 0.005 and < 0.0001, respectively; Fig. 3F). The results of qPCR showed that the copy number of EGFP transcripts transcribed from the cOVA locus was significantly higher than those transcribed from cROSA and cHIPP loci (*p* < 0.05 and *p* < 0.0001, respectively) (Fig. 3G).

Collectively, these data suggested that in the presence of a strong heterologous promoter, a non-GSH locus could support transcription higher than a GSH locus. So, it may be inferred that the transgene expression under a strong heterologous promoter is not entirely locus-dependent and is mostly promoter-dependent.

### Transgene Expression from the Weak Heterologous Promoter is Principally Locus-Dependent

**Fig. 4:**
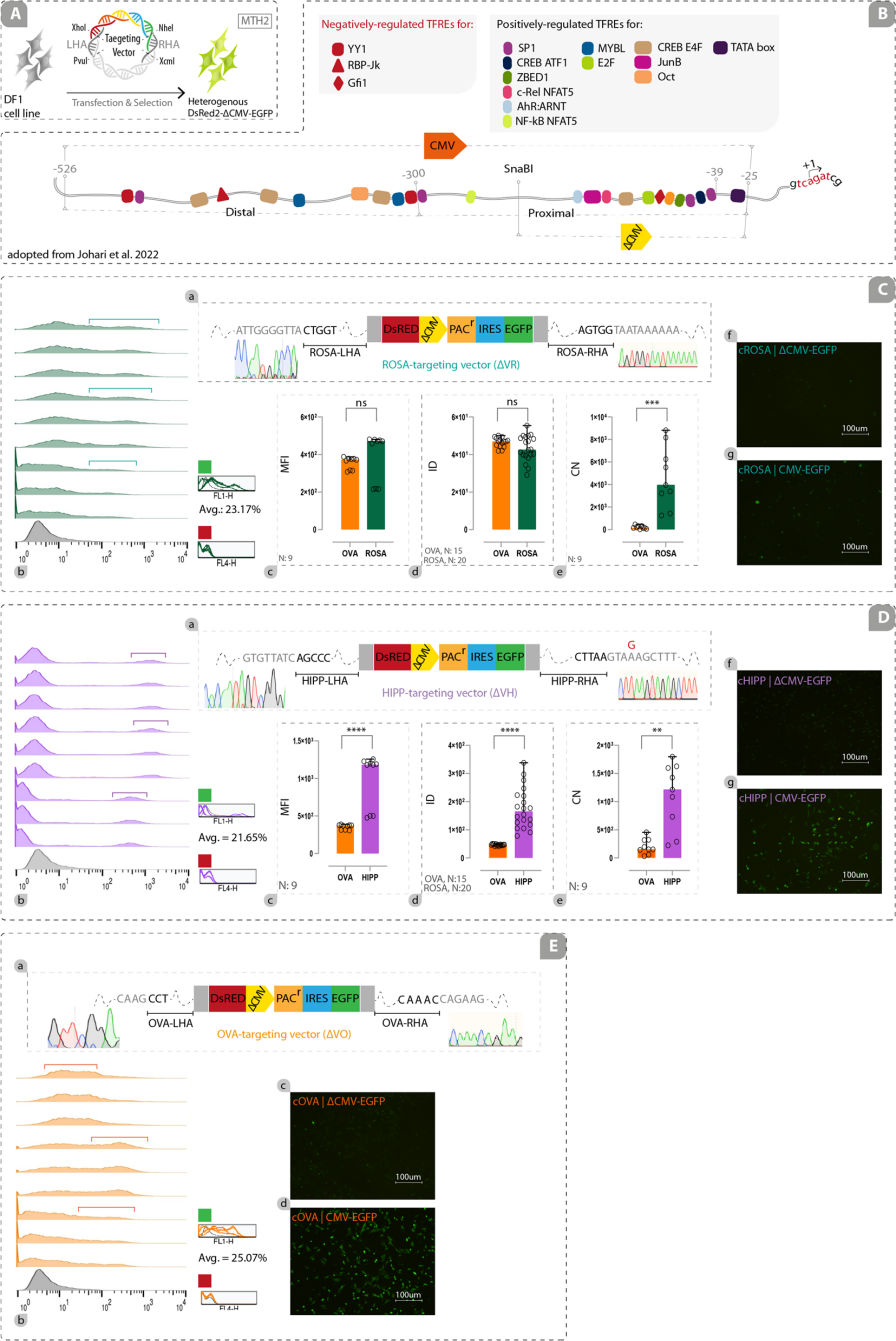
Transgene Expression from the weak heterologous promoter is principally locus-dependent. CRISPR-mediated integration of ∆CMV-driven EGFP and promoter-less DsRed2 in chicken predicted GSH loci and non-GSH locus was performed in DF1 cell lines. **A** Schematic depiction of ∆CMV-EGFP-expressing heterogeneous cell pools at the end of MTH2; **B** Schematic illustration of CMV and ∆CMV promoter as well as negatively- and positively-regulated transcription factor response elements (TFREs). C-a, D-a, E-a) CRISPR-mediated integration of DsRed2-∆CMV-EGFP in cROSA, cHIPP, and cOVA loci verified by 5’/3’ junction PCR, restriction enzyme digestion of the amplicons, and Sanger sequencing; C-b, D-b, E-b) flowcytometry results from EGFP-expressing cROSA, cHIPP, and cOVA cells have been achieved in three individual experiments (each in triplicates). Non-transfected cells have been used as a negative control. Average expression of EGFP for cROSA, cHIPP, and cOVA cell pools has been shown (green square). Expression of EGFP has been detected in the green channel. No expression signal has been detected in the red channel (red square); C–c, C-d, C-e, D-c, D-d, D-e) comparison of integrated density (ID) index, mean fluorescent intensity (MFI) index, and copy numbers (CN) of EGFP transcripts have been conducted among the main experimental groups (i.e., cROSA and cHIPP) against the control group (cOVA). C-f, D-f, E-c, C-g, D-g, E-d) Fluorescence microscope images of the cells expressing ∆CMV-driven EGFP and CMV-driven EGFP heterogeneously (Scale bar: 100um). ns: non-significant, ***: *p* < 0.005, and ****: *p* < 0.0001 are statistically significant. Avg.: The average expression of EGFP. N: Number

We expected that predicted GSH loci to support the elevated expression of a transgene under the control of a strong heterologous promoter. However, the EGFP expression from the non-GSH cOVA locus greatly outweighed the EGFP expression from the predicted GSH loci. Therefore, we assumed that the presence of a strong heterologous promoter unpredictably affects the expression of the integrated transgene. Thus, in the second preliminary study, we set out to evaluate the expression of EGFP under the control of a weak promoter integrated into the predicted GSH loci of cROSA and cHIPP, as well as the non-GSH cOVA locus (Additional file 7A; Fig. 4A, B). To this end, we generated three new targeting vectors named ∆VR, ∆VH, and ∆VO (Fig. 4B; C-a, D-a, E-a) in which EGFP was under the control of ∆CMV. Heterogenous cell pools harboring DsRed2-∆CMV-EGFP were cultured for two months. The 5’/3’ junction PCR (Additional file 9A-b, A-e, B-b, B-e, C-b, C-e), restriction enzyme digestion of the amplicons (Additional file 9A-c, B-c, C–c), and Sanger sequencing (Additional file 10A-b, B-b, C-b) were performed to verify knocked-in ∆VR, ∆VH, and ∆VO in the designated loci. In the absence of locus-specific gRNAs, 5’/3’ junction PCR did not verify knock-ins in the experimental groups (Additional file 9A-b, A-e, B-b, B-e, C-b, C-e).

**Fig. 5:**
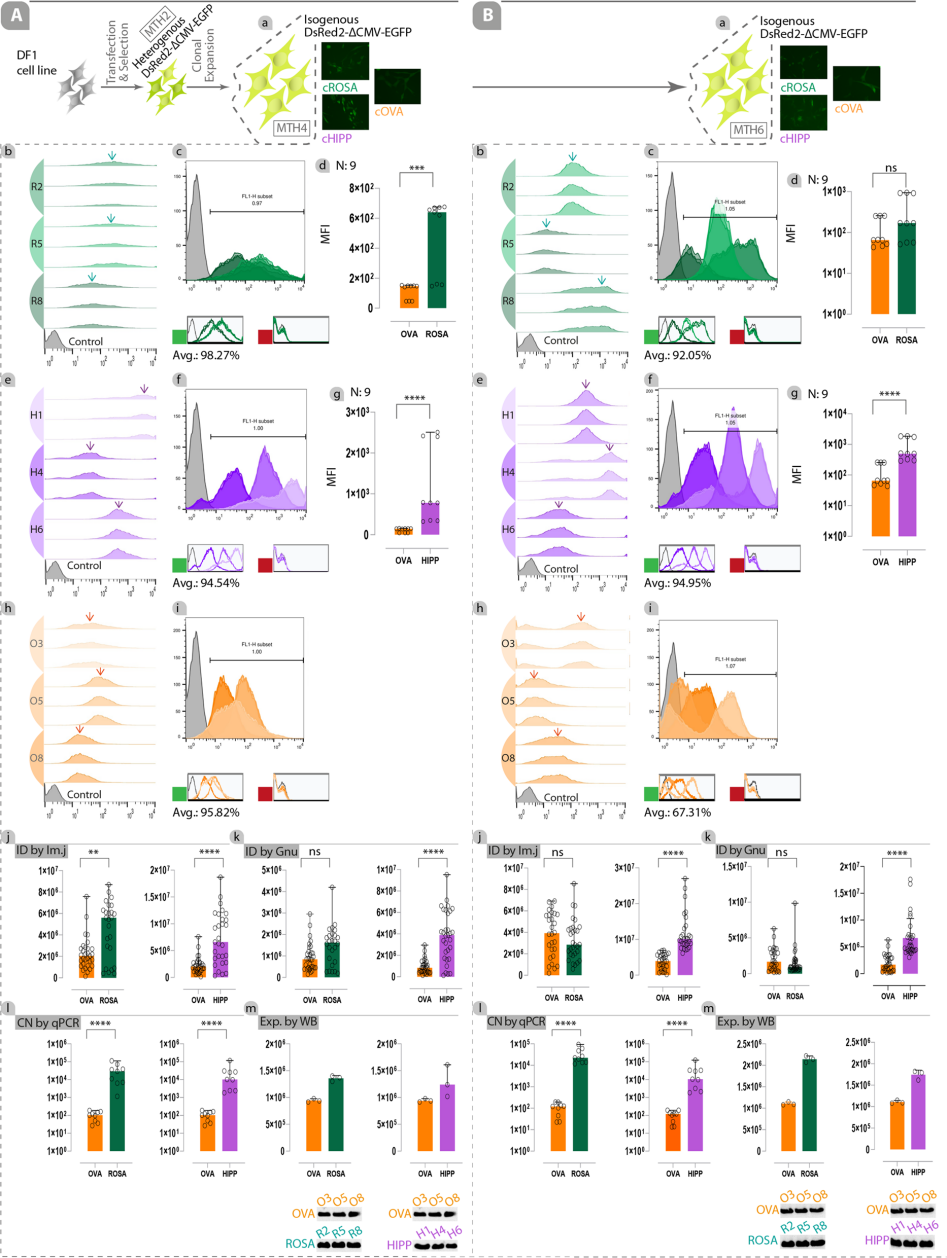
Transgene expression from the weak heterologous promoter is consistent and homogeneous in the potential GSH loci of isogenous cell clones. Clonally-expanded isogenous cells harboring the ∆CMV-driven EGFP in the potential GSH loci were able to consistently express the transgene. A-a, B-a) Schematic depiction of the process in which clonally-isolated cells were cultured for about six months. Offset (A-b, B-b for cROSA; A-e, B-e for cHIPP; A-h, B-h for cOVA at the end of MTH4 and MTH6) and overlay (A-c, B-c for cROSA; A-f, B-f for cHIPP; A-i, B-i for cOVA at the end of MTH4 and MTH6) illustrate the EGFP expression levels for correctly-targeted isogenous cell clones targeted at the cROSA (clones R2, R5, R8), cHIPP (clones H1, H4, H6), and cOVA (clones O3, O5, O8) loci. Shifting the peak to the right in the offsets shows an increase in the expression of EGFP (arrows show the high density of EGFP-positive cell clones). The MFI index in the cROSA (A-d, B-d) and cHIPP (A-g, B-g) clones with cOVA clones at the end of MTH4 and MTH6 were compared. Green squares show the average expression of EGFP for cROSA, cHIPP, and cOVA clones in the green channel. No expression signal was detected in the red channel (red square). The integrated density (ID) index was compared using the ImageJ (A-j, B-j) and GNUastro (A-k, B-k) software. The copy number of EGFP transcripts (A-l, B-l), and the expression levels of EGFP (A-m, B-m) were determined in the main experimental groups (i.e., cROSA and cHIPP) versus the control group (cOVA) at the end of MTH4 and MTH6. ns: non-significant, ***: *p* < 0.005, and ****: *p* < 0.0001 are statistically significant. Avg.: The average expression of EGFP. N: number. Integrated density by imageJ (ID by Im.J). Integrated density by GNUastro (ID by Gnu). Copy Number (CN) by qPCR. Expression by Western Blotting (Exp. by WB)

At the end of MTH2, 23.17%, 21.65%, and 25.07% of cells harboring the transgene in cROSA, cHIPP, and cOVA loci, were EGFP-positive, respectively (Fig. 4C-b, D-b, E-b). In contrast to the use of CMV, ∆CMV could improve MFI index and ID index in favor of GSH loci. MFI index of ∆CMV-EGFP inserted in the cHIPP locus was significantly higher than that for the ∆CMV-EGFP inserted in the cOVA locus (*p* < 0.0001; Fig. 4D-c), but there was no statistically significant difference between MFI of ∆CMV-EGFP inserted in the cROSA and cOVA loci (Fig. 4C–c). Highly variable levels of EGFP expression in heterogenous cell pools were observed, as demonstrated by wide histograms (Fig. 4C-b, D-b, E-b). Moreover, no expression of promoter-less DsRed2 was detected in all loci (red square), judged by flow cytometry (Fig. 4C-b, D-b, E-b). ID index was calculated by analyzing images captured from each locus (Additional file 14). The ID index findings supported MFI index (Fig. 4C-d, D-d). The copy number of transcripts from ∆CMV-driven EGFP inserted in the cROSA and cHIPP loci was significantly higher than those transcribed from the cOVA locus (*p* < 0.005 and *p* < 0.0001, respectively; Fig. 5C-e, D-e).

Comparison of EGFP expression and transcription status in heterogeneous cell pools harboring the CMV-driven EGFP or ∆CMV-driven EGFP integrated into designated loci confirmed that the strong activity of the CMV promoter has been significantly reduced when the promoter changed to ∆CMV (*p* < 0.0001), judged by MFI index, ID index, and qPCR (Additional file 15A, B, C). Also, fluorescence microscopy images showed a reduction in fluorescence intensity when the weak promoter was used (Fig. 4C-f, C-g, D-f, D-g, E-c, E-d). The only exception was the MFI results of the cHIPP locus, as there was no significant difference in the MFI index between CMV-driven EGFP and ∆CMV-driven EGFP (Additional file 15B-a). Overall, these results highlighted the beneficial effects of the weak heterologous promoter for evaluating and finding potential GSH loci.

### Transgene Expression from the Weak Heterologous Promoter is Consistent and Homogenous in the Potential GSH Loci of Isogenous Cell Clones

We reasoned that the expression of the transgene under the control of a weak promoter in the potential GSH loci might be more locus-dependent and homogenous in isogenous cell clones. To this end, isogenous cell clones were isolated from the heterogenous cell pools harboring ∆CMV-driven EGFP (integrated into GSH and non-GSH loci) which were in culture for more than 2 months (Additional file 7A). Furthermore, the R2, R5, and R8 clones (cROSA clones that contain DsRed2-∆ CMV-EGFP-IRES-PAC^r^ in the cROSA locus), the H1, H4, and H6 clones (cHIPP clones that contain the same cassette in the cHIPP locus), and the O3, O5, and O8 clones (cOVA clones that contain the same cassette in the cOVA locus) were expanded and analyzed at the end of MTH4 (Fig. 5A-a) and MTH6 (Fig. 5B-a). After isolation of single-cell clones by limit-diluting method (Additional file 8A), they were screened for bi- or mono-allelic knock-ins (Additional file 8B), and were subjected to 5’/3’ junction PCR (Additional file 8C) with further validation by restriction enzyme digestion (Additional file 8D) and Sanger sequencing (Additional file 10A-c, B-c, C–c). To confirm single-copy transgene knock-in, the copy number of EGFP transcripts transcribed from the GSH loci and non-GSH locus was determined (Additional file 8E).

Evaluation of the homogenous expression of EGFP in the correctly-targeted isogenous cell clones showed that cHIPP clones had highly uniform levels of EGFP expression compared to the cROSA clones, as demonstrated by narrow histograms in the offset graph (Fig. 5A-b, A-e, B-b, B-e). Although EGFP expression was homogeneous in the cOVA clones, transgene silencing occurred over time, judged by shifting the peak to the left in the offset graph from MTH4 to MTH6 (Fig. 5A-h, B-h). The average expression of EGFP (green square) in the MTH4 for cHIPP, cROSA, and cOVA were 98.27%, 94.54%, and 95.82%, respectively (Fig. 5A-c, A-f, A-i); while it was 92.05%, 94.95%, and 67.31% in the MTH6, respectively (Fig. 5B-c, B-f, B-i). Moreover, no expression of the promoter-less DsRed2 (red square) was detected in all loci during the six-month culture of these cells (Fig. 5A-c, A-f, A-i, B-c, B-f, B-i). Also, the results of our findings showed that integration of the transgene in the candidate GSH loci does not alter the morphology and doubling time of cells, either in targeted heterogeneous or in isogenous cells (Additional file 15D, E, F, G).

At the end of MTH4, the comparison of the MFI index of GSH loci with that of the non-GSH locus showed that the MFI index of cROSA clones Fig. 5A-d, *p* < 0.005) as well as cHIPP clones (Fig. 5A-g, *p* < 0.0001) were significantly higher than that of cOVA clones. At the end of MTH6, the same comparison was made and results showed that the cHIPP clones have maintained their superiority of transgene expression over cOVA clones and are consistently expressing the transgene (Fig. 5B-g), but cROSA clones showed reduced EGFP expression to almost near the expression level of that in cOVA clones (Fig. 5B-d). At the end of MTH4, analysis of captured images from each locus (Additional file 16) showed that both cROSA (*p* < 0.005) and cHIPP (*p* < 0.0001) clones had significantly higher ID index than cOVA clones (Fig. 5A-j) (analyzed by imageJ and GNUastro softwares). However, the comparison of this index between cROSA and cOVA clones showed no significant differences, judged by GNUastro software (Fig. 5A-k). qPCR results showed that the copy number of EGFP transcripts transcribed from the cROSA and cHIPP loci (*p* < 0.0001) was significantly higher than those from the cOVA locus (Fig. 5A-l). Moreover, western blot analyses confirmed that the expression levels of EGFP in cROSA and cHIPP clones were higher than those in cOVA clones (Fig. 5A-m and Additional file 17).

At the end of MTH6, results were similar to what was found in MTH4 (Fig. 5B-j, B-k, B-l, B-m). The only exception was that the ID index of cROSA clones was reduced compared with that in MTH4, and no significant differences with the ID index of cOVA clones were detected (Fig. 5B-j). The coefficient of variation (CV) of data extracted from ID index for each locus at the end of MTH4 and MTH6 was compared to evaluate the homogeneity of expression of the transgene (Additional file 15A-d, B-d, C-d). Among all loci, the cHIPP locus supported the homogenous expression of the knocked-in transgene more than the other loci. Although data showed that the cROSA locus can support the long-term and stable transgene expression better than the cOVA locus, CV values for both loci increased over time, indicating the heterogeneity in the expression of the transgene (Additional file 15A-d, B-d, C-d).

To determine whether isolated isogenous cell clones harbor mono-copy or multi-copies of the EGFP transgene in the genome, a standard curve was plotted using a serial dilution of the mix containing EGFP plasmid and the haploid equivalent of chicken genomic DNA (ratio 1:1). Ct values of isogenous cell clones indicated that EGFP transgene has been integrated into the genome of isogenous cell clones in a mono-copy manner (Additional file 8E).

Altogether, these results demonstrated that consistent and sustainable expression of a transgene could be achieved using weak promoters integrated into a GSH locus. Among evaluated GSH loci, the cHIPP locus supports the consistent and homogenous expression of the transgene better than cROSA locus.

## Discussion

This study set out to identify and evaluate novel GSH loci that can support a predictable, durable, and safe expression of desired genetic constructs in the chicken genome. First, we used a multi-omics bioinformatics pipeline to predict GSH loci (Fig. 1) in three individual experiments using DF1 cells (Additional file 7). Using this pipeline, potential GSH loci were selected from two regions: 7500 bp upstream of the THUMPD3 gene (consciously inside the LOC121106669 gene), and an 1100-bp intergenic region between the DRG1 and EIF4ENIF1 coding genes (Fig. 2). Then, we performed functional experiments by integrating the transgene into the predicted GSH loci as well as a non-GSH locus. In the first preliminary study, results revealed that EGFP expression from the CMV promoter was not entirely locus-dependent (Fig. 3). The evidence from the second preliminary study suggested that EGFP expression drived by ∆CMV promoter was principally locus-dependent (Fig. 4). Moreover, the isogenous cell clones with ∆CMV-driven EGFP integration were derived. Long-term transgene expression in GSH loci from the ∆CMV promoter was locus-dependent, consistent, and homogenous in isogenous cell clones (Fig. 5).

For this study, an intergenic region between the DRG1 and EIF4ENIF1 genes and inside of an unknown gene located upstream of the THUMPD3 gene was chosen as a potential GSH locus to insert the transgene. Also, the first intron of the cOVA gene was chosen as a non-GSH locus. To insert the transgene, three individual gRNAs were designed for each locus. Those gRNAs that had high rank, high GC contents and low self-complementary were used in the CRISPR/Cas9-based HDR knock-ins. The current data highlight the importance of gRNA’s GC content, as cleavage activities remarkably decreased with increasing GC content. Hence, gRNAs with high GC content were used to avoid the high activity of Cas9 and possible off-target effects [52]. Also, avoiding self-complementary should be considered for choosing an effective gRNA [53]. Since, in this study, the genetically engineered cells were achieved by either antibiotic selection or limit diluting method, there was no need to apply highly-active gRNA for increasing knock-in efficiency.

It has been revealed that the genomic and epigenomic interactions occur within the individual TADs [54], or specifically inside the sub-TAD regions [55]. To avoid unwanted/irregular interactions among the transgene integrated into the targeted locus and the genes located in the same sub-TAD, it is preferred to insert the transgene at least in a gene-poor sub-TAD [8]. It has been proposed to analyze the TAD/sub-TAD arrangements for evaluating GSH loci [7]. Unfortunately, sub-TAD data has not been registered in the chickens so far, but TAD data is available [48]. Hence, we located the coordinates of cROSA, cHIPP, and cOVA loci in the genome of chicken according to the previously-reported TAD data [48]. However, we found that the cROSA, cHIPP, and cOVA loci are located in the TADs in which 15, 19, and 16 genes are annotated, respectively. Due to the lack of sub-TAD data, we could not exactly specify the number of genes in the same sub-TAD where those loci are located. Although studies related to the gene therapy emphasize that linear and three-dimensional distance from a GSH locus to neighboring genes, far-distance genes, and regulatory elements should be evaluated [8], this can be ruled out for biotechnological applications including protein manufacturing [24] and generating genetically-engineered animals [5, 19, 56]. Similar to the mouse HIPP (H11) locus [5], the cHIPP locus is located in a transcriptionally active intergenic region, judged by RNAseq data of flanked genes adopted from gene expression atlas (https://www.ebi.ac.uk/gxa/home). Despite the cROSA locus which has been chosen near the highly-active THUMPD3 gene, unpredictable/fluctuated expression of the knocked-in transgene from the cROSA locus seems to occur due to the integration site of the transgene inside an unknown gene.

For an accurate functional evaluation of potential GSH loci, the durable/stable and homogenous expression of a mono-copy transgene should be met. The stable expression of the mono-copy transgene is preferable to multi-copy transgenes [31], or concatemers of transgenic DNA [28]. Also, it has been found that while the expression of a multi-copy transgene is 1.5 times higher than a single-copy transgene, the coefficient of variation for the multi-copy transgene is 6 times higher than that for a single-copy transgene, indicating the heterogeneity of expression [30]. Hence, isogenous cell clones harboring a mono-copy transgene in the potential GSH locus are required. These kinds of cells provide low clonal variation and homogenous expression of the transgene [10, 57, 58]. In this study, a dual reporter cassette was inserted into the cROSA and cHIPP GSH loci as well as into the cOVA non-GSH locus. Expression of CMV- or ∆CMV-driven EGFP and promoter-less DsRed2 were evaluated. Durable and stable EGFP expressions were used to determine the reliability of the expression from the locus, and the lack of DsRed2 expression specified that there weren’t any cis- or trans-regulations on the transgene. These findings are in line with the previous study reported by Ruan et al. [19].

Several studies have used strong heterologous promoters to explore whether a predicted locus is GSH [3, 5, 6, 10, 14, 21, 24, 26, 59]. Although the stable expression of the transgene (more than 3 months in the cell lines and at least 1 generation for transgenic animals) has been achieved using strong promoters, it is uneasy to determine whether this durable expression is locus-dependent or promoter-dependent [3, 5, 6, 10, 21, 24, 26]. In the first preliminary study, we began the functional analysis of GSH loci using a strong heterologous promoter and tested the expression status of CMV-driven EGFP inserted in both the non-GSH and potential GSH loci at the end of MTH2 in the continuously-cultured heterogenous cell pools (Additional file 7B). The results showed that the expression of CMV-driven EGFP integrated into the cOVA non-GSH locus outweighs to those integrated into the cROSA and cHIPP loci. This raises two questions; is this due to the conformational changes of the insertion site for the non-GSH locus that provides the ease of accessibility of transcription factors, and improves the EGFP expression? or that negative transcription factors may have a rapid occupancy rate on the CMV’s transcription factor regulatory elements (TFREs) at the GSH loci, leading to the epigenetic silencing?

Since the priority of EGFP expression was not observed in the non-GSH locus compared to GSH loci when we used the ΔCMV promoter (weak promoter), it seems unlikely that the change in the conformation of the region upon transgene insertion is the reason for this phenomenon. Epigenetic features of the target locus are remodeled upon transgene insertion [4]. If this happened, it might be the same for all loci. Due to the susceptibility of the CMV promotor to methylation [60, 61], it seems that epigenetic silencing of the CMV promoter in transcriptionally active loci rapidly outweighs that in the non-GSH locus. It has been found that transcriptionally active loci are highly accessible to the transcription factors compared with nucleosome-dense loci [62, 63]. We speculated that the occupancy of transcriptionally active loci by both activators and repressors is the same and it might be higher in GSH loci than those in the non-GSH locus. Therefore, repression of CMV in GSH loci may rapidly occur, indicating much more recruitment of negative regulatory elements. It has been demonstrated that CMV is variably repressed in the mouse ROSA locus in several tissues [29]. Also, the insertion of CAG-driven transgene in the AAVS1 locus has resulted in transgene silencing both mono-allelically and bi-allelically [64]. Both studies are indicating the inefficiency of a strong promoter for the stable/durable expression of a transgene.

To alleviate the epigenetic silencing, we used the ∆CMV promoter. Context-specific transcriptional performance and cell-specific expression of CMV promoter have been identified both in vivo [65, 66] and in vitro [67, 68]. Due to the discrepancy between the cell’s repertoire of endogenous transcription factors, interpretation of the results among the studies should be performed with caution [32]. It has been found that all positive TFREs are located at the proximal region (i.e., approximately -300 to -36, relative to the TSS) and negative TFREs reside at the distal part of the CMV (i.e., approximately -550 to -300). YY1, RBP-Jκ, and Gfi1 are the main negative transcription factors as their over-expression results in CMV repression in mouse fibroblast cells [69–71]. Accordingly, we removed the distal part as well as a part of proximal CMV (i.e., approximately -550 to -245) and kept the sequence from -245 to + 1 (named ∆CMV). Reduced expression of EGFP observed in DF1 cells when ∆CMV was used might be due to the removal of most of the positive TFREs located in -550 to -245. This finding is in line with the finding of another study that the use of -239 to + 48 led to more than 2 times reduction of EGFP expression in HEK cells compared with full-length CMV [32].

In the second preliminary study that was conducted in parallel with the first one, the expression status of ∆CMV-driven EGFP was evaluated. Interestingly, the results changed in favor of GSH loci, as the expression of ∆CMV-driven EGFP integrated into the cROSA and cHIPP loci outweighed the expression from those integrated in the cOVA non-GSH locus. Although the expression/fluorescence of EGFP was reduced, the removal of the negative TFREs from CMV might result in the predictable/expected behavior of GSH loci. Also, significant variability of expression may be masked when EGFP is used as a reporter due to its stability and saturation at high levels [30]. Therefore, we concluded that isogenous cell clones harboring a transgene controlled by weak promoters may resolve this issue and would be the best choice for evaluating GSH loci.

From these two preliminary studies, we inferred that the use of ∆CMV would be beneficial for evaluating GSH loci. Also, for further validation, homogenous expression and less clonal variation were required [10, 57]. Hence, isogenous cell clones harboring mono-copy/mono-allelic DsRed2-∆ CMV-EGFP integrated into these three loci were isolated. Stable/durable EGFP expression and the possible silencing of EGFP were evaluated over time. About 28% reduction of EGFP expression was seen in the cOVA locus, judged by flow cytometry. No expression reduction of EGFP was observed for GSH loci from the end of MTH4 to MTH6 in the 6-month continuously-cultured isogenous cell clones. This is in line with the studies in which the expression of integrated EGFP in AAVS1 and HIPP loci was stable from passage 0 to 30 [10] and over 3 months [3, 24], respectively. In these studies, EGFP was controlled by CAG or CMV promoters. So, this is unclear whether durable expression in these studies is promoter-dependent or locus-dependent. In another study, EGFP containing cassettes controlled by viral origin (SFFV) or cellular origin (PGK and EF1A with or without intron) promoters were integrated into AAVS1 and CCR5 loci [4]. Flow cytometry analyses showed that the strong SSFV and EF1A (with intron) promoters maintained high EGFP expression in both loci at the same level over 3 months, but the EGFP expression in the CCR5 locus started to reduce when relatively weak promoters including PGK and EF1A (without intron) were used. Since the CCR5 locus is susceptible to silencing by cis-acting DNA sequences, this result demonstrated that the transgene expression is locus-dependent when weak cellular origin promoters were used [4]. Also, expression of the EGFP controlled by SSFV or EF1A (with intron) promoters for about 5 weeks that was stably integrated into the intergenic, intronic, and enhancer loci generated the same results [30] as reported by Lombardo et al. [4].

We observed heterogeneity of EGFP expression among isogenous cell clones. This can be related to genetic variability and genome instability in studied immortalized cell lines that can lead to heterogenous expression even in different isogenous cell clones [3]. Also, expression variability of the transgene in isogenous cell clones has been reported to be influenced by genome plasticity, stochasticity in biochemical reactions, and global interconnected cellular constraints (reviewed in [11]). These instabilities may occur in the long-term cultured isogenous cell clones due to the loss or rearrangement of transgenes [72] or changes in epigenetic regulations of DNA or histones [73]. Inevitable intrinsic clonal cell diversity and noisy gene expression may be uncontrollable [11]. Interestingly, a high level of clonal variations was observed even in the master cell lines in which the same locus was targeted by several transgenes [57]. Hence, heterogenous EGFP expression among isogenous cell clones is inevitable. Thus, to evaluate new GSH loci for transgenic technology applications, establishing isogenous cell clones with low clonal variations would be beneficial over the use of master cell clones.

In a previous study, “attP landing pads” were specifically inserted in the GSH loci of the host cell by CRISPR/Cas technology, and single cells harboring these pads were sorted to generate “master cell lines” for inserting the transgene using the RMCE method into these landing pads [10]. Despite having genome plasticity and fewer position effects, these cell lines may lower clonal cell diversity and heterogenous productivity. These cells might be very beneficial for manufacturing recombinant proteins, but they may not be suitable for the evaluation of GSH loci. The organization of TADs and epigenetic marks may be very similar in these cells, avoiding them showing different expression profiles, leading to false positive results. Hence, it is preferred to use individual isogenic cell clones that are isolated from heterogenous cell pools, providing more realistic conditions for the evaluation of GSH candidate loci.

## Conclusions

In this study, two novel genomic safe harbor loci named cHIPP and cROSA were found in the chicken genome. We reasoned that the evaluation of reliable GSH loci should be performed by weakened promoter in parallel with non- GSH loci. In contrast to our expectation, the transgene expression under the control of a full-length CMV promoter in a non-GSH locus outperformed the one integrated into the GSH locus. We inferred that if a strong promoter was used to evaluate GSH loci, transgene expression would be promoter-dependent not locus-dependent. So, evaluation of GSH loci harboring a transgene controlled by a strong promoter will not be reliable. Hence, we used weakened form of CMV to evaluate GSH loci. Our findings showed that in order to precisely evaluate the GSH loci by weakened form of CMV, a non-GSH locus should be used in parallel with potential GSH loci. Using a non-GSH locus in parallel with GSH locus can precisely decipher whether transgene expression is locus-dependent or promoter-dependent. In fact, the expression of transgene should be locus-dependent when GSH loci are being investigated.

## Supplementary Information

**Additional file 1.** Chicken HiC data.

**Additional file 2. **Coordinates of chicken defined TAD boundaries.

**Additional file 3. **Coordinates of the chicken annotated genes.

**Additional file 4.** 5’ and 3’ homology arm (HA) sequences spanning the sgRNA target sites.

**Additional file 5.** Oligonucleotides used in this study.

**Additional file 6.** cROSA, cHIPP, and cOVA gRNA were designed by CHOPCHOP online software.

**Additional file 7.** Different cell lines generated and used in this study.

**Additional file 8.** Isolating isogenous cell clones, verification of correctly-targeted clones, and confirmation of mono-allelic and single copy knocked-in transgenes in GSH and non-GSH loci.

**Additional file 9.** Verification of CRISPR-mediated knock-ins in the chicken GSH loci and non-GSH locus.

**Additional file 10.** Sanger sequencing analysis of 5’- and 3’-flanking junctions of correctly-targeted GSH loci and non-GSH locus.

**Additional file 11.** GNUastro command lines used for analysis of images.

**Additional file 12.** The coordinates of cROSA, cHIPP, and cOVA loci as well as flanking genes have been visualized by JUICEBOX online software (the coordinate system of the map corresponds to the genome version GalGal5).

**Additional file 13.** Images of heterogenous cell pools harboring CMV-EGFP for analyzing by ImageJ software.

**Additional file 14.** Images of heterogenous cell pools harboring ΔCMV-EGFP for analyzing by ImageJ software.

**Additional file 15.** Comparison of EGFP expression levels in heterogenous cell pools and isogenous cell clones harboring CMV-driven EGFP or ∆CMV-driven EGFP and doubling time and morphology of targeted cells.

**Additional file 16.** Images of isogenous cell clones harboring the DsRed2-ΔCMV-EGFP transgene.

**Additional file 17.** Western blot analysis for evaluating the expression of the EGFP protein.

## Data Availability

All data generated or analysed during this study are included in this published article (and its supplementary information files). Also, All data are available from the corresponding author upon reasonable request.

## Abbreviations

GSH: Genomic Safe Harbor
cHIPP: Chicken HIPP-like
cROSA: Chicken ROSA-like
ΔCMV: Weakened Form of the CMV Promoter
TFREs: Transcription Factor Response Elements
TAD: Topologically Associated Domain
TPM: Transcript per Million
HA: Homology Arm
gDNA: Genomic DNA
MTH4: Month Four
MTH6: Month Six
MFI: Mean Fluorescence Intensity
CN: Copy Number
ID: Integrated Density

## References

[1] Oleg E. Tolmachov, Subkhankulova T, Tolmachov T. Silencing of Transgene Expression: A Gene Therapy Perspective. Gene Ther - Tools Potential Appl. 2013. 10.5772/53379.

[2] Dehdilani N, Yousefi Taemeh S, Goshayeshi L, Dehghani H (2022). Genetically Engineered Birds; pre-CRISPR and CRISPR era†. Biol Reprod.

[3] Aznauryan E, Yermanos A, Kinzina E (2022). Discovery and Validation of Human Genomic Safe Harbor Sites for Gene and Cell Therapies. Cell Rep Methods..

[4] Lombardo A, Cesana D, Genovese P (2011). Site-Specific Integration and Tailoring of Cassette Design for Sustainable Gene Transfer. Nat Methods.

[5] Tasic B, Hippenmeyer S, Wang C (2011). Site-Specific Integrase-Mediated Transgenesis in Mice via Pronuclear Injection. Proc Natl Acad Sci U S A.

[6] Ghahfarokhi MK, Dormiani K, Mohammadi A, Jafarpour F, Nasr-Esfahani MH (2017). Blastocyst Formation Rate and Transgene Expression are Associated with Gene Insertion into Safe and Non-Safe Harbors in the Cattle Genome. Sci Rep.

[7] Pellenz S, Phelps M, Tang W (2019). New Human Chromosomal Sites with “Safe Harbor” Potential for Targeted Transgene Insertion. Hum Gene Ther.

[8] Shrestha D, Bag A, Wu R (2022). Genomics and Epigenetics Guided Identification of Tissue-Specific Genomic Safe Harbors. Genome Biol.

[9] Gu B, Posfai E, Rossant J (2018). Efficient Generation of Targeted Large Insertions by Microinjection into Two-Cell-Stage Mouse Embryos. Nat Biotechnol.

[10] Shin S, Kim SH, Shin SW (2020). Comprehensive Analysis of Genomic Safe Harbors as Target Sites for Stable Expression of the Heterologous Gene in HEK293 Cells. ACS Synth Biol.

[11] Lee JS, Kildegaard HF, Lewis NE, Lee GM (2019). Mitigating Clonal Variation in Recombinant Mammalian Cell Lines. Trends Biotechnol.

[12] Irion S, Luche H, Gadue P, Fehling HJ, Kennedy M, Keller G (2007). Identification and Targeting of the ROSA26 Locus in Human Embryonic Stem Cells. Nat Biotechnol.

[13] Kimura Y, Shofuda T, Higuchi Y (2019). Human Genomic Safe Harbors and the Suicide Gene-Based Safeguard System for iPSC-Based Cell Therapy. Stem Cells Transl Med.

[14] Ma L, Wang Y, Wang H, et al. Screen and Verification for Transgene Integration Sites in Pigs. 2018:1–11. 10.1038/s41598-018-24481-1. 10.1038/s41598-018-24481-1 PMC5943519 29743638

[15] Chen Y, Mao S, Liu B, et al. Novel Mosaic Mice with Diverse Applications. bioRxiv. 2020:2020.03.21.001388. 10.1101/2020.03.21.001388.

[16] Kobayashi T, Kato-Itoh M, Yamaguchi T (2012). Identification of Rat Rosa26 Locus Enables Generation of Knock-in Rat Lines Ubiquitously Expressing tdTomato. Stem Cells Dev.

[17] Tasic B, Miyamichi K, Hippenmeyer S (2012). Extensions of MADM (Mosaic Analysis with Double Markers) in Mice. PLoS one.

[18] Yang D, Song J, Zhang J (2016). Identification and Characterization of Rabbit ROSA26 for Gene Knock-in and Stable Reporter Gene Expression. Sci Rep.

[19] Ruan J, Li H, Xu K, Wu T, Wei J, Zhou R. Highly Efficient CRISPR / Cas9- Mediated Transgene Knockin at the H11 Locus in Pigs. Nat Publ Gr. 2015:1–10. 10.1038/srep14253. 10.1038/srep14253 PMC4585612 26381350

[20] Wang M, Sun Z, Zou Z (2018). Efficient Targeted Integration Into the Bovine Rosa26 Locus Using TALENs. Sci Rep.

[21] Wu M, Wei C, Lian Z, et al. Rosa26 -Targeted Sheep Gene Knock-in via CRISPR-Cas9 System. Nat Publ Gr. 2016:1–7. 10.1038/srep24360. 10.1038/srep24360 PMC4827023 27063570

[22] Li X, Yang Y, Bu L (2014). Rosa26-Targeted Swine Models for Stable Gene Over-Expression and Cre-Mediated Lineage Tracing. Cell Res.

[23] Zhu F, Gamboa M, Farruggio AP (2014). DICE, an Efficient System for Iterative Genomic Editing in Human Pluripotent Stem Cells. Nucleic Acids Res.

[24] Chi X, Zheng Q, Jiang R, Chen-Tsai RY, Kong LJ (2019). A System for Site-Specific Integration of Transgenes in Mammalian Cells. PLoS one.

[25] Gaidukov L, Wroblewska L, Teague B (2018). A Multi-Landing Pad DNA Integration Platform for Mammalian Cell Engineering. Nucleic Acids Res.

[26] Perez-Pinera P, Ousterout DG, Brown MT, Gersbach CA (2012). Gene Targeting to the ROSA26 Locus Directed by Engineered Zinc Finger Nucleases. Nucleic Acids Res.

[27] Hockemeyer D, Soldner F, Beard C (2009). Efficient Targeting of Expressed and Silent Genes in Human ESCs and iPSCs Using Zinc-Finger Nucleases. Nat Biotechnol.

[28] Remy S, Tesson L, Menoret S (2014). Efficient Gene Targeting by Homology-Directed Repair in Rat Zygotes Using TALE Nucleases. Genome Res.

[29] Rizzi N, Rebecchi M, Levandis G, Ciana P, Maggi A (2017). Identification of Novel Loci for the Generation of Reporter Mice. Nucleic Acids Res.

[30] Eyquem J, Poirot L, Galetto R, Scharenberg AM, Smith J (2013). Characterization of Three Loci for Homologous Gene Targeting and Transgene Expression. Biotechnol Bioeng.

[31] Liu T, Hu Y, Guo S (2018). Identification and Characterization of MYH9 Locus for High Efficient Gene Knock-in and Stable Expression in Mouse Embryonic Stem Cells. PLoS One.

[32] Johari YB, Scarrott JM, Pohle TH (2022). Engineering of the CMV Promoter for Controlled Expression of Recombinant Genes in HEK293 Cells. Biotechnol J.

[33] DeKelver RC, Choi VM, Moehle EA (2010). Functional Genomics, Proteomics, and Regulatory DNA Analysis in Isogenic Settings Using Zinc Finger Nuclease-Driven Transgenesis Into a Safe Harbor Locus in the Human Genome. Genome Res.

[34] Kong Q, Hai T, Ma J (2014). Rosa26 Locus Supports Tissue-Specific Promoter Driving Transgene Expression Specifically in Pig. PLoS one.

[35] Li G, Zhang X, Wang H (2020). CRISPR/Cas9-Mediated Integration of Large Transgene into Pig CEP112 Locus. G3 (Bethesda).

[36] Stanford WL, Cohn JB, Cordes SP, Lunenfeld S (2001). Gene-Trap Mutagenesis: Past, Present and Beyond. Nat Rev Genet.

[37] Papapetrou EP, Lee G, Malani N (2011). Genomic Safe Harbors Permit High β -Globin Transgene Expression in Thalassemia Induced Pluripotent Stem Cells. Nat Biotechnol.

[38] Miyata Y, Tokumoto S, Arai T (2022). Identification of Genomic Safe Harbors in the Anhydrobiotic Cell Line, Pv11. Genes (Basel).

[39] Lee ES, Moon S, Abu-Bonsrah KD (2019). Programmable Nuclease-Based Integration into Novel Extragenic Genomic Safe Harbor Identified from Korean Population-Based CNV Analysis. Mol Ther Oncolytics.

[40] Sadelain M, Papapetrou EP, Bushman FD (2011). Safe Harbours for the Integration of New DNA in the Human Genome. Nat Rev Cancer.

[41] Hilliard W, Lee KH (2021). Systematic Identification of Safe Harbor Regions in the CHO Genome Through a Comprehensive Epigenome Analysis. Biotechnol Bioeng.

[42] Park CY, Sung JJ, Cho SR, Kim J, Kim DW (2019). Universal Correction of Blood Coagulation Factor VIII in Patient-Derived Induced Pluripotent Stem Cells Using CRISPR/Cas9. Stem Cell Rep.

[43] Li YS, Meng RR, Chen X (2019). Generation of H11-albumin-rtTA Transgenic Mice: A Tool for Inducible Gene Expression in the Liver. G3 (Bethesda).

[44] Waters PD, Patel HR, Ruiz-Herrera A (2021). Microchromosomes are Building Blocks of Bird, Reptile, and Mammal Chromosomes. Proc Natl Acad Sci.

[45] Ordovás L, Boon R, Pistoni M (2015). Efficient Recombinase-Mediated Cassette Exchange in hPSCs to Study the Hepatocyte Lineage Reveals AAVS1 Locus-Mediated Transgene Inhibition. Stem Cell Rep.

[46] Strathdee D, Ibbotson H, Grant SGN (2006). Expression of Transgenes Targeted to the Gt(ROSA)26Sor Locus is Orientation Dependent. PLoS one.

[47] Nyabi O, Naessens M, Haigh K (2009). Efficient Mouse Transgenesis Using Gateway-compatible ROSA26 Locus Targeting Vectors and F1 Hybrid ES Cells. Nucleic Acids Res.

[48] Fishman V, Battulin N, Nuriddinov M (2019). 3D organization of Chicken Genome Demonstrates Evolutionary Conservation of Topologically Associated Domains and Highlights Unique Architecture of Erythrocytes’ Chromatin. Nucleic Acids Res.

[49] Merkin J, Russell C, Chen P, Burge CB (2012). Evolutionary Dynamics of Gene and Isoform Regulation in Mammalian Tissues. Science.

[50] Barbosa-Morais NL, Irimia M, Pan Q (2012). The Evolutionary Landscape of Alternative Splicing in Vertebrate Species. Science.

[51] Engreitz JM, Haines JE, Perez EM (2016). Local Regulation of Gene Expression by lncRNA Promoters, Transcription and Splicing. Nature.

[52] Malik A, Gul A, Munir F (2021). Evaluating the Cleavage Efficacy of CRISPR-Cas9 sgRNAs Targeting Ineffective Regions of Arabidopsis Thaliana Genome. PeerJ.

[53] Beeber D, Chain FJ (2020). crispRdesignR: A Versatile Guide RNA Design Package in R for CRISPR/Cas9 Applications. J genomics.

[54] Rao SSP, Huntley MH, Durand NC (2014). A 3D Map of the Human Genome at Kilobase Resolution Reveals Principles of Chromatin Looping. Cell.

[55] Hnisz D, Day DS, Young RA (2016). Insulated Neighborhoods: Structural and Functional Units of Mammalian Gene Control. Cell.

[56] Browning J, Rooney M, Hams E (2020). Highly Efficient CRISPR-Targeting of the Murine Hipp11 Intergenic Region Supports Inducible Human Transgene Expression. Mol Biol Rep.

[57] Grav LM, Sergeeva D, Lee JS (2018). Minimizing Clonal Variation during Mammalian Cell Line Engineering for Improved Systems Biology Data Generation. ACS Synth Biol.

[58] O’Brien SA, Lee K, Fu HY (2018). Single Copy Transgene Integration in a Transcriptionally Active Site for Recombinant Protein Synthesis. Biotechnol J.

[59] Ménoret S, De Cian A, Tesson L (2015). Homology-Directed Repair in Rodent Zygotes Using Cas9 and TALEN Engineered Proteins. Sci Rep.

[60] Brooks AR, Harkins RN, Wang P, Qian HS, Liu P, Rubanyi GM (2004). Transcriptional Silencing is Associated with Extensive Methylation of the CMV Promoter Following Adenoviral Gene Delivery to Muscle. J Gene Med.

[61] Moritz B, Becker PB, Göpfert U (2015). CMV Promoter Mutants with a Reduced Propensity to Productivity Loss in CHO Cells. Sci Rep.

[62] Boeger H, Griesenbeck J, Strattan JS, Kornberg RD (2003). Nucleosomes Unfold Completely at a Transcriptionally Active Promoter. Mol Cell.

[63] Klemm SL, Shipony Z, Greenleaf WJ (2019). Chromatin Accessibility and the Regulatory Epigenome. Nat Rev Genet.

[64] Bhagwan JR, Collins E, Mosqueira D (2019). Variable Expression and Silencing of CRISPR-Cas9 Targeted Transgenes Identifies the AAVS1 Locus as Not an Entirely Safe Harbour. F1000Research.

[65] Mella-Alvarado V, Gautier A, Le Gac F, Lareyre JJ (2013). Tissue and Cell-specific Transcriptional Activity of the Human Cytomegalovirus Immediate Early Gene Promoter (UL123) in Zebrafish. Gene Expr Patterns.

[66] Vasey DB, Lillico SG, Sang HM, King TJ, Whitelaw CBA (2009). CMV Enhancer-Promoter is Preferentially Active in Exocrine Cells in Vivo. Transgenic Res.

[67] Qin JY, Zhang L, Clift KL (2010). Systematic Comparison of Constitutive Promoters and the Doxycycline-Inducible Promoter. PLoS one.

[68] Xia W, Bringmann P, McClary J (2006). High Levels of Protein Expression Using Different Mammalian CMV Promoters in Several Cell Lines. Protein Expr Purif.

[69] Zweidler-Mckay PA, Grimes HL, Flubacher MM, Tsichlis PN (1996). Gfi-1 Encodes a Nuclear Zinc Finger Protein that Binds DNA and Functions as a Transcriptional Repressor. Mol Cell Biol.

[70] Liu XF, Yan S, Abecassis M, Hummel M (2008). Establishment of Murine Cytomegalovirus Latency in Vivo is Associated with Changes in Histone Modifications and Recruitment of Transcriptional Repressors to the Major Immediate-Early Promoter. J Virol.

[71] Liu R, Baillie J, Sissons JG, Sinclair JH (1994). The Transcription Factor YY1 Binds to Negative Regulatory Elements in the Human Cytomegalovirus Major Immediate Early Enhancer/Promoter and Mediates Repression in Non-Permissive Cells. Nucleic Acids Res.

[72] Kim M, O’Callaghan PM, Droms KA, James DC (2011). A Mechanistic Understanding of Production Instability in CHO Cell Lines Expressing Recombinant Monoclonal Antibodies. Biotechnol Bioeng.

[73] O’Callaghan PM, Racher AJ. Building a Cell Culture Process with Stable Foundations: Searching for Certainty in an Uncertain World. In: Al-Rubeai M, ed. Animal Cell Culture. Springer International Publishing, Springer, Cham; 2015:373-406. 10.1007/978-3-319-10320-4_12.

