# Supplementary material for: Integrating Omics and CRISPR Technology for Identification and Verification of Genomic Safe Harbor Loci in the Chicken Genome
Source: Biol Proced Online. 2023 Jun 24;25:18. doi: 10.1186/s12575-023-00210-5 (PMC10290409; doi:10.1186/s12575-023-00210-5)
Supplement: Supplementary file 1 — Additional file 1. Chicken HiC data. [file 12575_2023_210_MOESM1_ESM.docx]

**Additional File 1.** Chicken HiC data

The files are accessible from the link below:

<https://genedev.bionet.nsc.ru/ftp/by_Species/Vertebrates/Chicken/hic/>
