# Supplementary material for: Integrating Omics and CRISPR Technology for Identification and Verification of Genomic Safe Harbor Loci in the Chicken Genome
Source: Biol Proced Online. 2023 Jun 24;25:18. doi: 10.1186/s12575-023-00210-5 (PMC10290409; doi:10.1186/s12575-023-00210-5)
Supplement: Supplementary file 4 — Additional file 4. 5’ and 3’ homology arm (HA) sequences spanning the sgRNA target sites. [file 12575_2023_210_MOESM4_ESM.docx]

**Additional File 4. 5’ and 3’** **homology arm (HA) sequences spanning the sgRNA target sites**

**Left homology arm corresponding to cHIPP-like locus: 520bp**

agccctaggggaggtcctgttatccaagtgcagcagtttgactgtactagcagtttaaaggctgatcttgacagccagggtttaagagtaaggaattaaatactaatataccagatgcacatgcagcagctgttgaaccctgctctgccactaagcagcacctagaggaagggtggatgagcctctgttcatagcttctccactaccctctgctgtacccttacagtcatgtagctgttaaggaaaatttaagtgcttgaactaaaccagaatcaaagcttagtagcaacctctgggaaaagttccttatacactccaatgtccctacttccatttttgtaggagtcactgctagcagctcacaactgtgatagaactctgactgaaacaaaaaacttagctttattcacttctattgtgaaacagaagtactgctttaagagctgccaactttccttgtacaactaagtttggagaagtcaaataagcaggaaattcttttaagaaacctccaaagctt

**Right homology arm corresponding to cHIPP-like locus: 306bp**

atctagcttgcttccactagtataaacaattgaaaaagtgactactcaaaatcaaaacctagcacctcttccataactgcaggactattaatagccttgaagcattctatcaggaacatctgaaattgaggcagccagagctcacattttatgtccttgagttctgccttccaagctatggttctccagcctctgctttttggcttgttctaaattagagcagagtaatttcagctgtgtgaactcttccaatttgaagtcagcacaggcatcaaagcttcttttgccattgtgaaaagtacttaa

**Left homology arm corresponding to cROSA-like locus: 501bp**

ctggtcaagaaaaggggaacaactgggaaaagtggtgctttcctagaactgacttccccaaagcgttctgaccacaaacaactcacaactgagcaaacacttccctcccaggctgtgtgcctggcgtgctgctgctaacgtggggggctttgctctgcagctgcccccaggccagggcatgggcacagcattgcatgtgtgctgcacgaggccttcacaaagcaccaggtacagcttgctatgagccaaactggagaagcagtgagcacacgtgagtaatgggtgggatttttgtaccctctgaaataaagcctcacagatttgaattgatgtgaatgactgtatcaacagtaaatcaaatccggtactccaaaagggtgggtagctcaggccataacaaaatgggtttgattatctgtcacgttgcataattccaaacttacaaggcttgattttctggcttcggtgttttgcagtggaggacaaattgccatcatgcac

**Right homology arm corresponding to cROSA-like locus: 491bp**

actcactgcgacggctgcagtgcatcgtgtgagctgtgcttgatgaaagaggtctgcttctaacagccacgtagacatggatatagaacaaaagaattaataagttggtgaaacagtgagaacatttaaatctgcagcttttcccagactctgttccctgtggcatcgcacagcttccccagcttcacttcagaatacctccccagctcagtgccaggtacctggggctcacatgaattccggattccccatggttaattctacagactgtgtccaatggtacctaacccaggaggcacctcccctcaccctccagaaaacacattgtagaatttgttaagtaatgacagacacttcgtcaaacagaccattcatctcaacatgtgaaccagaaggcgcagaaggtttgtttctaaacatgtaaaaagtccatgcagaccagcggtgcttctggagactctattggcagctgtgggttgggaatggagtgg

**Left homology arm corresponding to cOVA locus: 556bp**

cctctgctttctcatatatctgtccaaacctaaagtttactgaaatttgctctttgaatttccagttttgcaagcctatcagattgtgttttaatcagaggtactgaaaagtatcaatgaattctagctttcactgaacaaaaatatgtagaggcaactggcttctgggacagtttgctacccaaaagacaactgaatgcaaatacataaatagatttatgaatatggttttgaacatgcacatgagaggtggatatagcaacagacacattaccacagaattactttaaaactacttgttaacatttaattgcctaaaaactgctcgtaatttactgttgtagcctaccatagagtaccctgcatggtactatgtacagcattccatccttacattttcactgttctgctgtttgctctagacaactcagagttcaccatgggctccatcggtgcagcaagcatggaattttgttttgatgtattcaaggagctcaaagtccaccatgccaatgagaacatcttctactgccccattgccatcatgtcagctcta

**Right homology arm corresponding to cOVA locus: 526bp**

gtgcaaaagacagcaccaggacacagataaataaggtgagcctacagttaaagattaaaacctttgccctgctcaatggagccacagcacttaattgtatgataatgtcccttggaaactgcatagctcagaggctgaaaatctgaaaccagagttatctaaaagtgtggccacctccaactcccagagtgttacccaaatgcactagctagaaatcttgaaactggattgcataacttctttttgtcataaccattatttcagctactattattttcaattacaggttgttcgctttgataaacttccaggattcggagacagtattgaagctcaggtacagaaataatttcacctccttctctatgtccctttcctctggaagcaaaatacagcagatgaagcaatctcttagctgttccaagccctctctgatgagcagctagtgctctgcatccagcagttgggagaacactgttcataagaacagagaaaaagaaggaagtaacaggggattcagaacaaa
