# Supplementary material for: Integrating Omics and CRISPR Technology for Identification and Verification of Genomic Safe Harbor Loci in the Chicken Genome
Source: Biol Proced Online. 2023 Jun 24;25:18. doi: 10.1186/s12575-023-00210-5 (PMC10290409; doi:10.1186/s12575-023-00210-5)
Supplement: Supplementary file 5 — Additional file 5. Oligonucleotides used in this study. [file 12575_2023_210_MOESM5_ESM.docx]

| **Primer code** | **Primer original name** | **Primer sequence (5'->3')** |
| --- | --- | --- |
| P1 | Ch-ROSA-LHA_F | CTGGTCAAGAAAAGGGGAACAACTGGG |
| P2 | Ch-ROSA-LHA_R | GTTTGGCCCCTGGGCTGTCAG |
| P3 | RE_Ch-ROSA-LHA_F | CCCAAGCTTGGGTCGCGATCGCGACTGGTCAAGAAAAGGGGAACAACTGGG |
| P4 | RE_Ch-ROSA-LHA_R | CCGGATATCCGGCCCCTTAAGGGGGTTTGGCCCCTGGGCTGTCAG |
| P5 | Ch-ROSA-RHA_F | ACTCACTGCGACGGCTGCAGTGC |
| P6 | Ch-ROSA-RHA_R | CCACTCCATTCCCAACCCACAGCTGC |
| P7 | RE_Ch-ROSA-RHA_F | CCCAAGCTTGGGCTAGCTAGCTAGACTCACTGCGACGGCTGCAGTGC |
| P8 | RE_Ch-ROSA-RHA_R | CCGGATATCCGGCCACTCCATTCCCAACCCACAGCTGC |
| P9 | LHA-H11-4017-HOM-F | CATGCATTAGTTCGCGATCGAGCCCTAGGGGAGGTCCTG |
| P10 | LHA-H11-4017-HOM-R | TGGCGACCGGTACCCTCGAGAAGAATTTCCTGCTTATTTGACTTCTCC |
| P11 | RHA-H11-4017-HOM-F | CTTTCTAGGGTTAAGCTAGCCTTCCACTAGTATAAACAATTG |
| P12 | RHA-H11-4017-HOM-R | TGGTGCCACCTATGTTGTGGAGAAATAAAACTGCTCTCCC |
| P13 | Ch-5-LHA_F | CCTCTGCTTTCTCATATATCTGTCC |
| P14 | Ch-5-LHA_R | TAGAGCTGACATGATGGCAATG |
| P15 | RE_Ch-5-LHA_F | CCCAAGCTTGGGTCGCGATCGCGACCTCTGCTTTCTCATATATCTGTCC |
| P16 | RE_Ch-5-LHA_R | CCGGATATCCGGCCCCTTAAGGGGTAGAGCTGACATGATGGCAATG |
| P17 | Ch-5-RHA_F | GTGCAAAAGACAGCACCAGGAC |
| P18 | Ch-5-RHA_R | TTTGTTCTGAATCCCCTGTTACTTCC |
| P19 | RE_Ch-5-RHA_F | CCCAAGCTTGGGCTAGCTAGCTAGGTGCAAAAGACAGCACCAGGAC |
| P20 | RE_Ch-5-RHA_R | CCGGATATCCGGTTTGTTCTGAATCCCCTGTTACTTCC |
| P21 | EGFP-Realtime/Fwd | AAGCTGACCCTGAAGTTCATCTGC |
| P22 | eGFP-Rv | GATGGTGCGCTCCTGGAC |
| P23 | Beta actin For2 | GAGAAGATGACACAGATC |
| P24 | Beta actin Rev2 | CAGAGTCCATCACAATAC |
| VS1 | Line1-Fw | GGGTTTATTTTATTAGGGAGTGTTAGA |
| VS2 | 3'TR-F | CCTCGATATACAGACCGATAAAACACATGCG |
| GS1 | Fw LAR | CAGAACAGAGGGAGCTCTATTTCCT |
| GS2 | RosaLike-Conf-Rv | CACCTATACTTGCTGCTGCACC |
| GS3 | Fw LAH | ACAGCCAGACTTGCTCTTTCCTAAG |
| GS4 | H11-conf | CTGCATATTATACTTCTAAGTCGTC |
| GS5 | Fw LAO | TACAATAGTTGTACAGTTCAGCTC |
| GS6 | RV RAO | TGAACGAATAAACATCATTTGGTTTG |
| GS7 | Rv RAH | GTGTCCTTCCATTTGTTTCTAAGTC |
