# Supplementary material for: Integrating Omics and CRISPR Technology for Identification and Verification of Genomic Safe Harbor Loci in the Chicken Genome
Source: Biol Proced Online. 2023 Jun 24;25:18. doi: 10.1186/s12575-023-00210-5 (PMC10290409; doi:10.1186/s12575-023-00210-5)
Supplement: Supplementary file 6 — Additional file 6. cROSA, cHIPP, and cOVA gRNA were designed by CHOPCHOP online software. [file 12575_2023_210_MOESM6_ESM.zip › (additional file 6) Legend - Proof version_ESM.docx]

**Additional File 6.** cROSA, cHIPP, and cOVA gRNA were designed by CHOPCHOP online software.

A-a) Cyan arrow shows the position and orientation of gRNAs specific for cROSA locus. A-b) Selected gRNA with the details specific for cROSA. A-c) The position of LHA and RHA as well as selected gRNA relative to the predicted cROSA locus and flanked genes. B-a) Purple arrow shows the position and orientation of gRNAs specific for cHIPP locus. B-b) Selected gRNA with the details specific for cHIPP. B-c) The position of LHA and RHA as well as selected gRNA relative to the predicted cHIPP locus and flanked genes. C-a) Orange arrow shows the position and orientation of gRNAs specific for cOVA locus. C-b) Selected gRNA with the details specific for cOVA. C-c) The position of LHA and RHA as well as selected gRNA relative to the predicted cOVA locus and flanked genes.
