# Supplementary figures and images for: Integrating Omics and CRISPR Technology for Identification and Verification of Genomic Safe Harbor Loci in the Chicken Genome
Source: Biol Proced Online. 2023 Jun 24;25:18. doi: 10.1186/s12575-023-00210-5 (PMC10290409; doi:10.1186/s12575-023-00210-5)

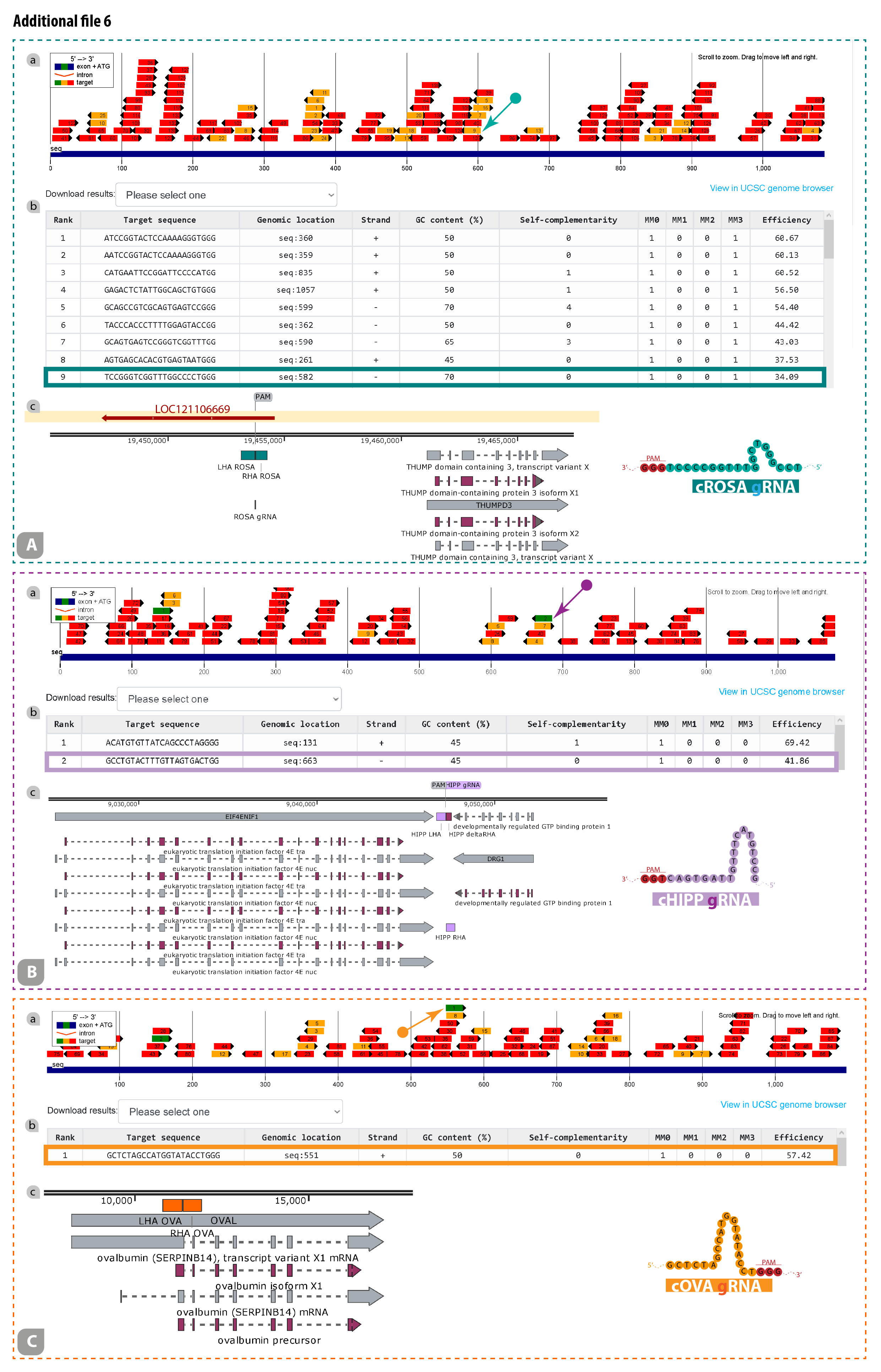

Supplement: Supplementary file 6 — Additional file 6. cROSA, cHIPP, and cOVA gRNA were designed by CHOPCHOP online software. [file 12575_2023_210_MOESM6_ESM.zip › (additional file 6) Proof version-01_ESM.tif]

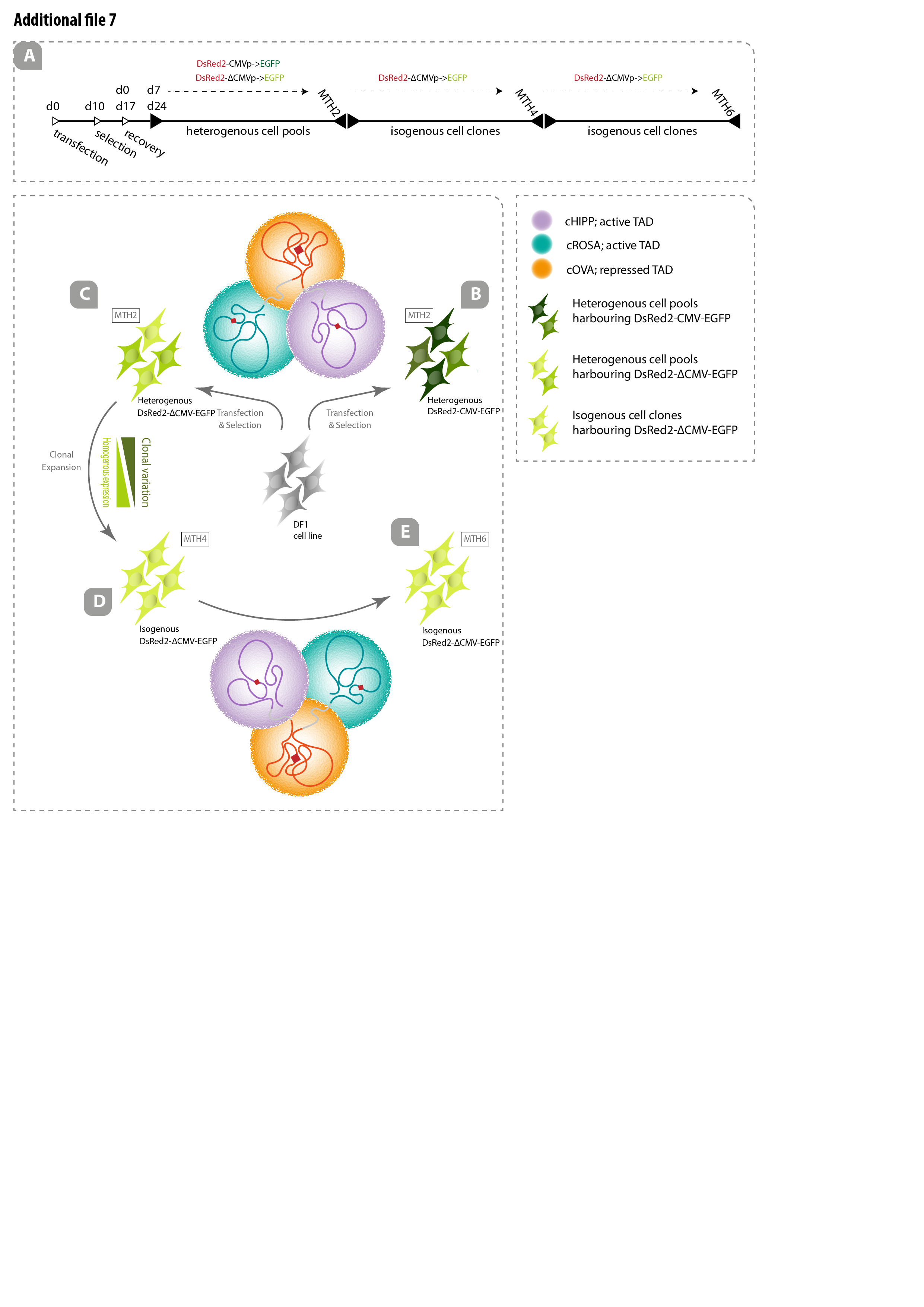

Supplement: Supplementary file 7 — Additional file 7. Different cell lines generated and used in this study. [file 12575_2023_210_MOESM7_ESM.zip › (additional file 7) Proof version-01_ESM.tif]

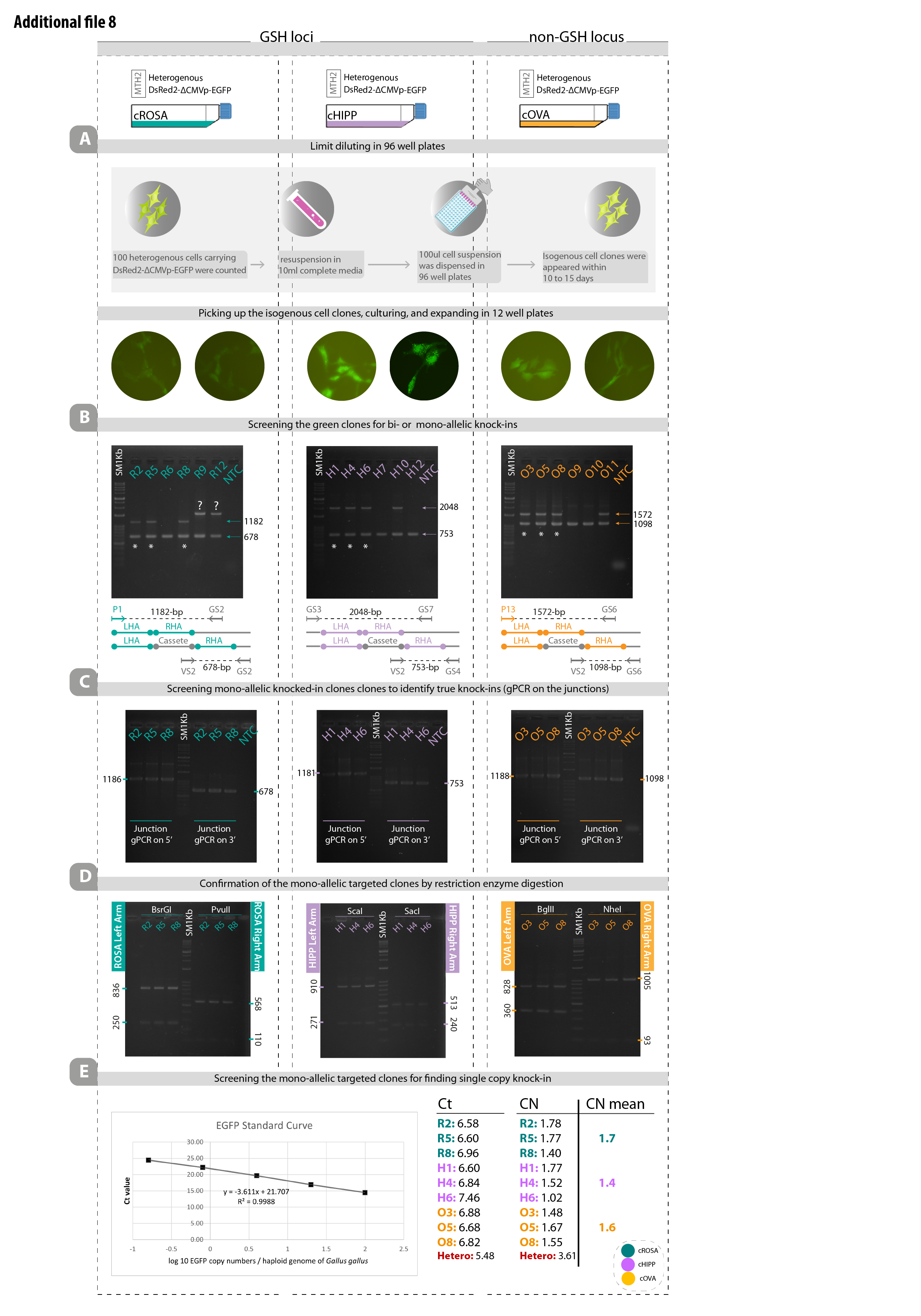

Supplement: Supplementary file 8 — Additional file 8. Isolating isogenous cell clones, verification of correctly-targeted clones, and confirmation of mono-allelic and single copy knocked-in transgenes in GSH and non-GSH loci. [file 12575_2023_210_MOESM8_ESM.zip › (additional file 8) Proof version-01_ESM.tif]

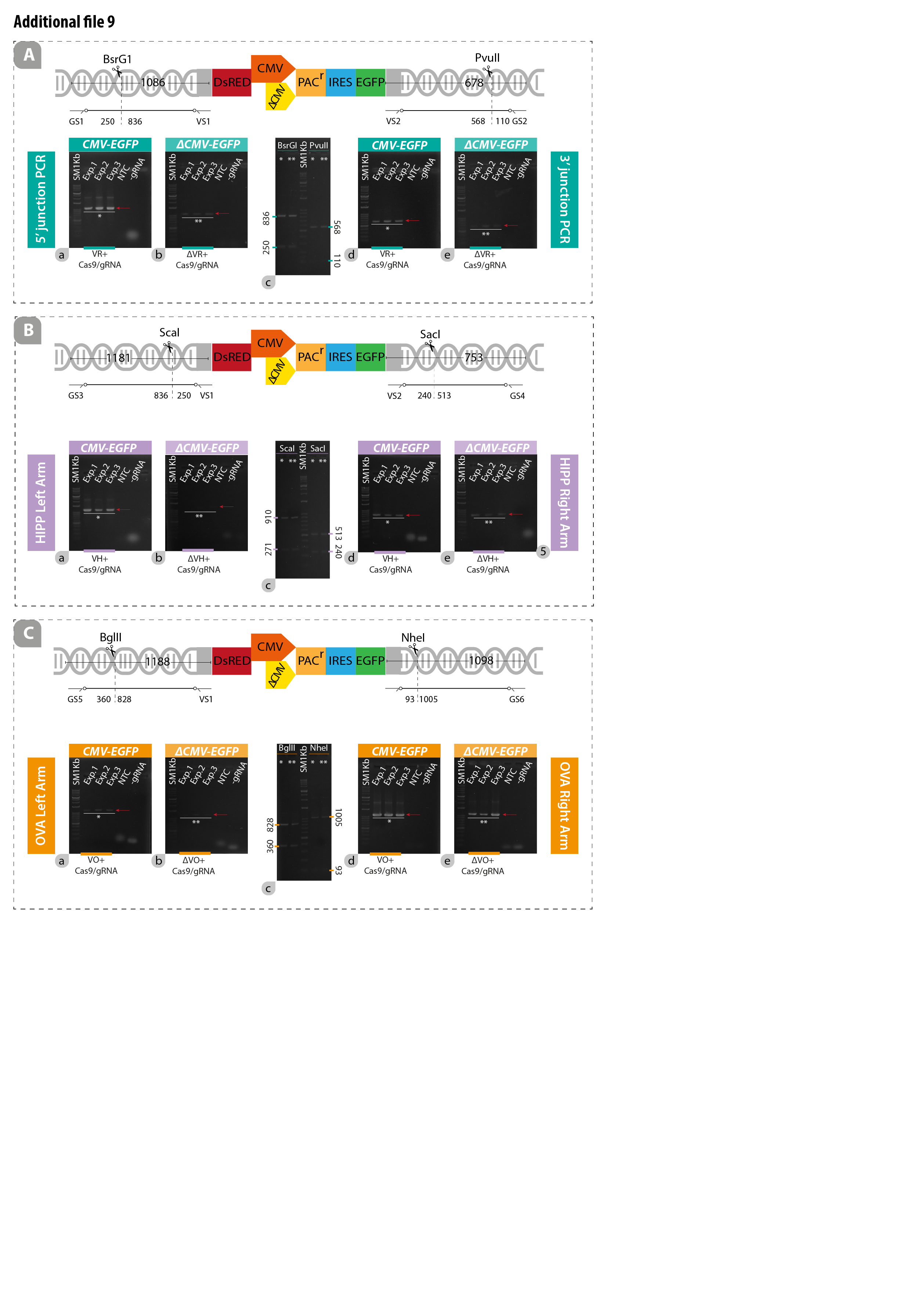

Supplement: Supplementary file 9 — Additional file 9. Verification of CRISPR-mediated knock-ins in the chicken GSH loci and non-GSH locus. [file 12575_2023_210_MOESM9_ESM.zip › (additional file 9) Proof version-01_ESM.tif]

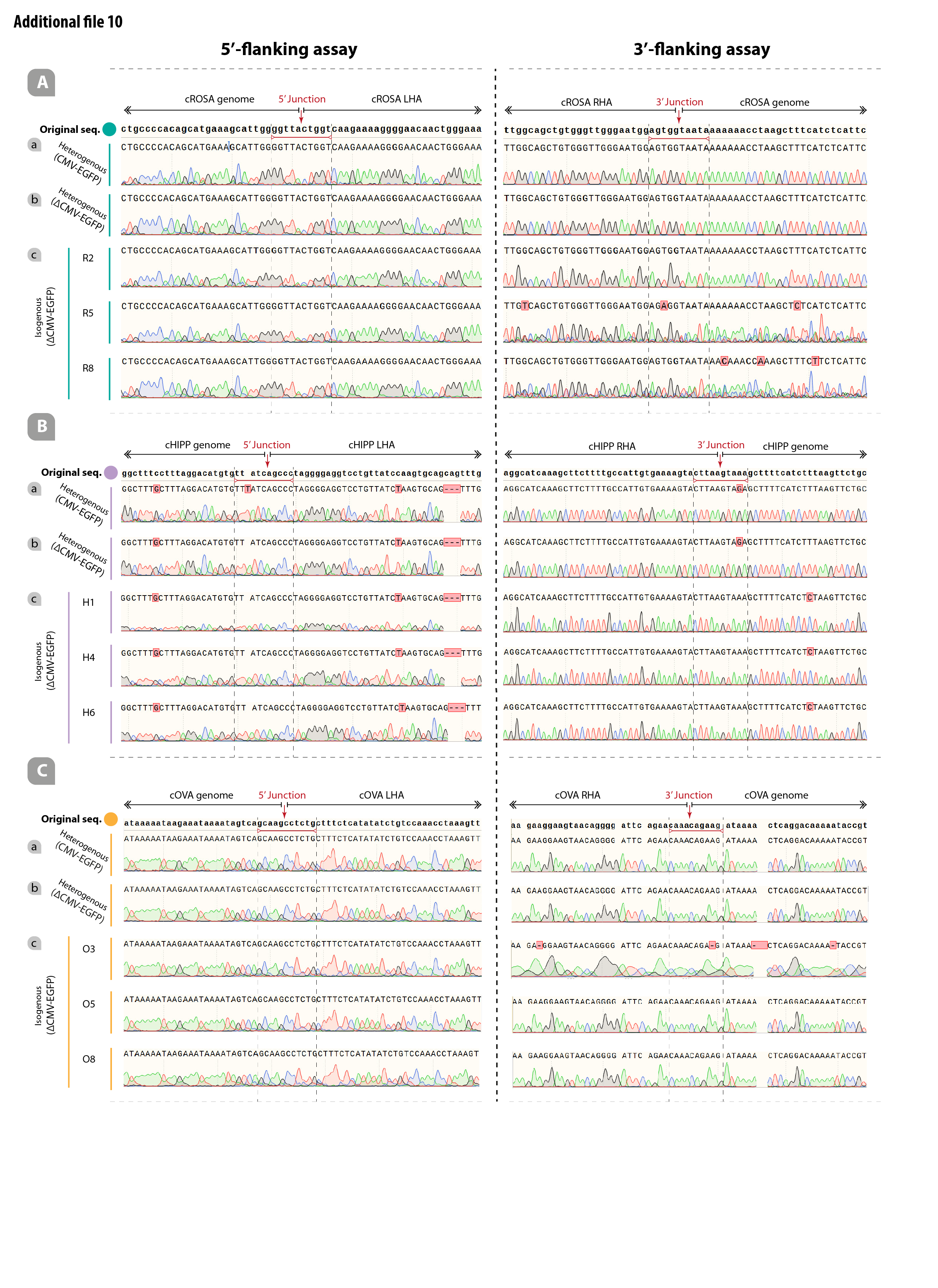

Supplement: Supplementary file 10 — Additional file 10. Sanger sequencing analysis of 5’- and 3’-flanking junctions of correctly-targeted GSH loci and non-GSH locus. [file 12575_2023_210_MOESM10_ESM.zip › (additional file 10) Proof version-01_ESM.tif]

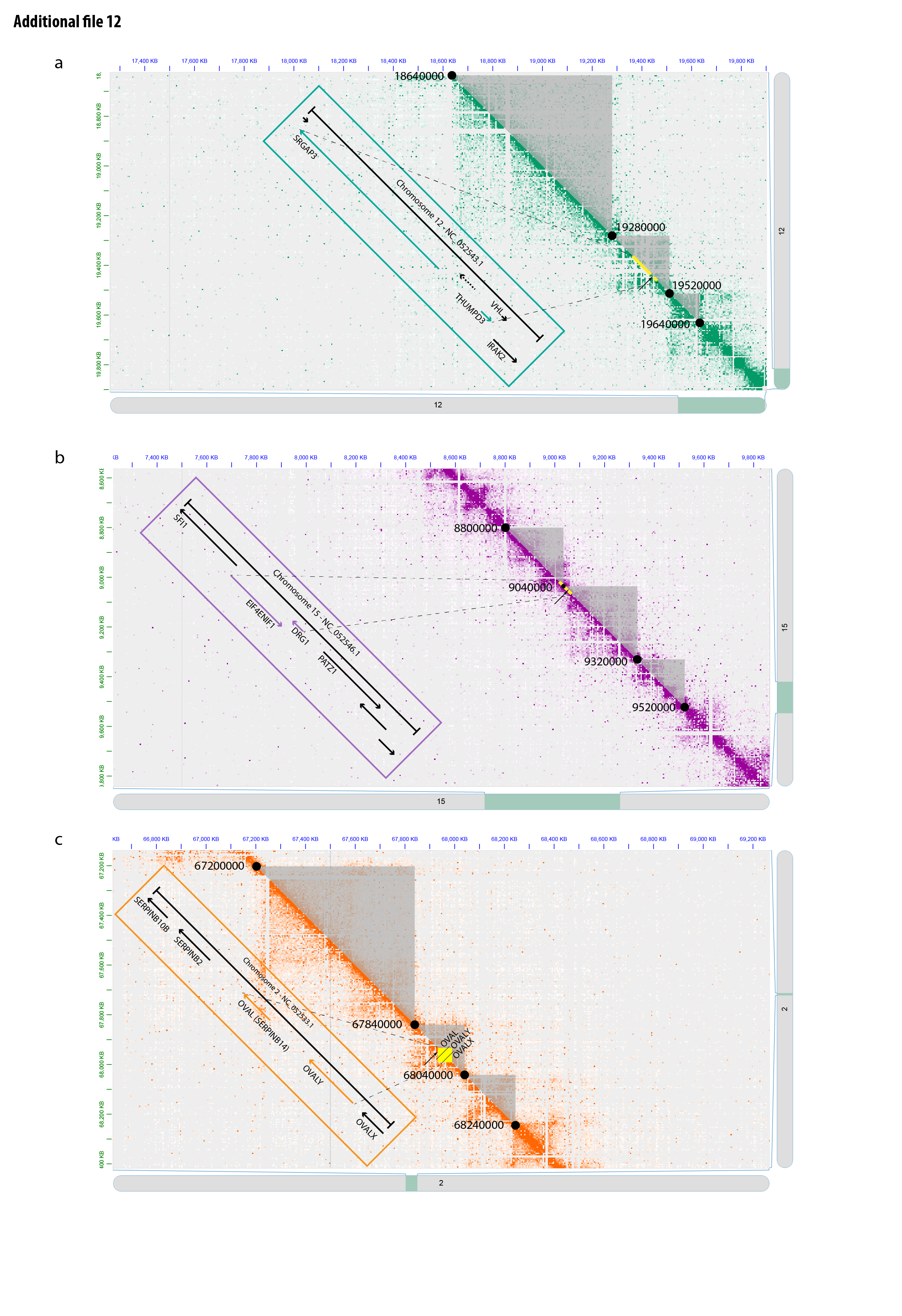

Supplement: Supplementary file 12 — Additional file 12. The coordinates of cROSA, cHIPP, and cOVA loci as well as flanking genes have been visualized by JUICEBOX online software (the coordinate system of the map corresponds to the genome version GalGal5). [file 12575_2023_210_MOESM12_ESM.zip › (additional file 12) Proof version-01_ESM.tif]

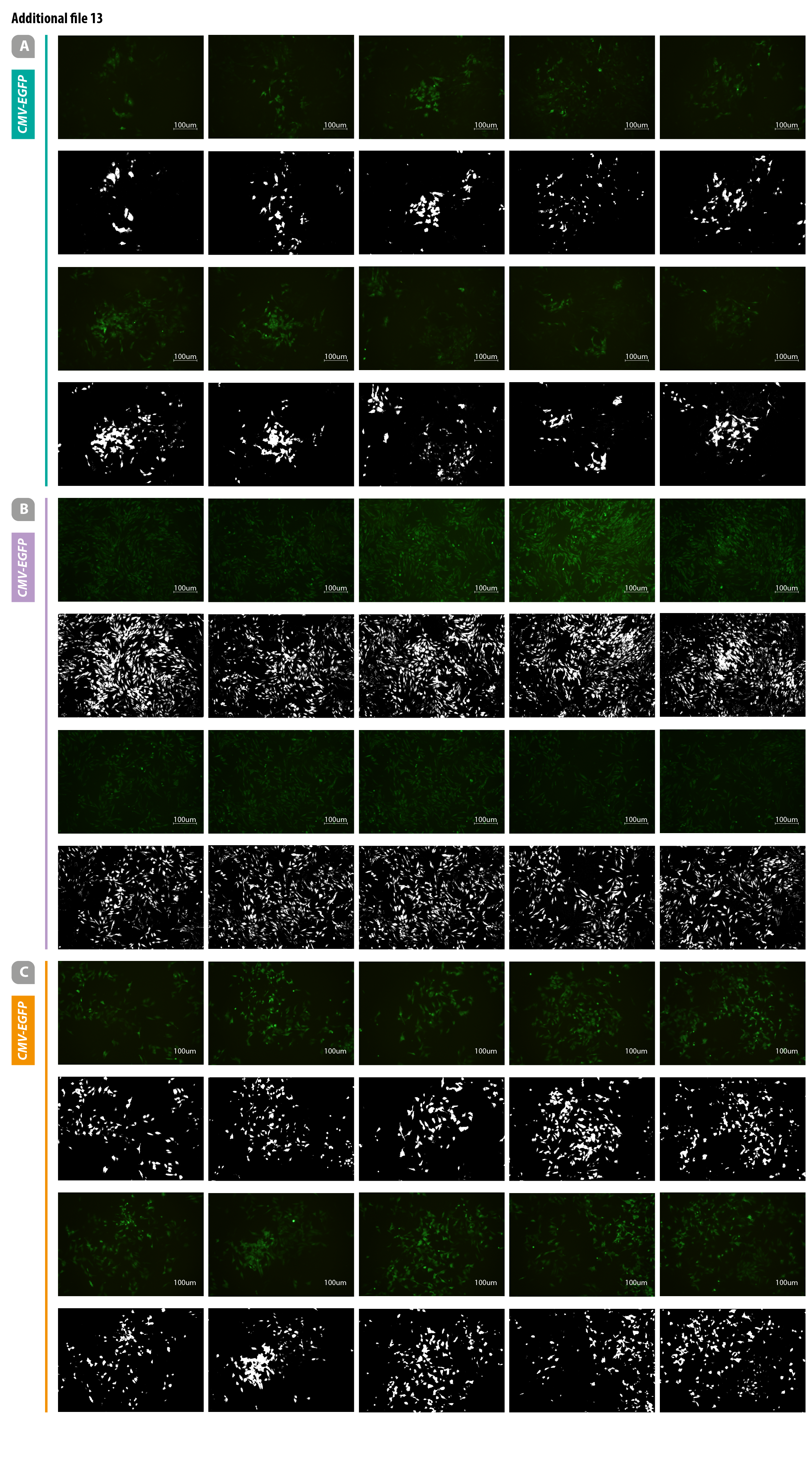

Supplement: Supplementary file 13 — Additional file 13. Images of heterogenous cell pools harboring CMV-EGFP for analyzing by ImageJ software. [file 12575_2023_210_MOESM13_ESM.zip › (additional file 13) Proof version-01_ESM.tif]

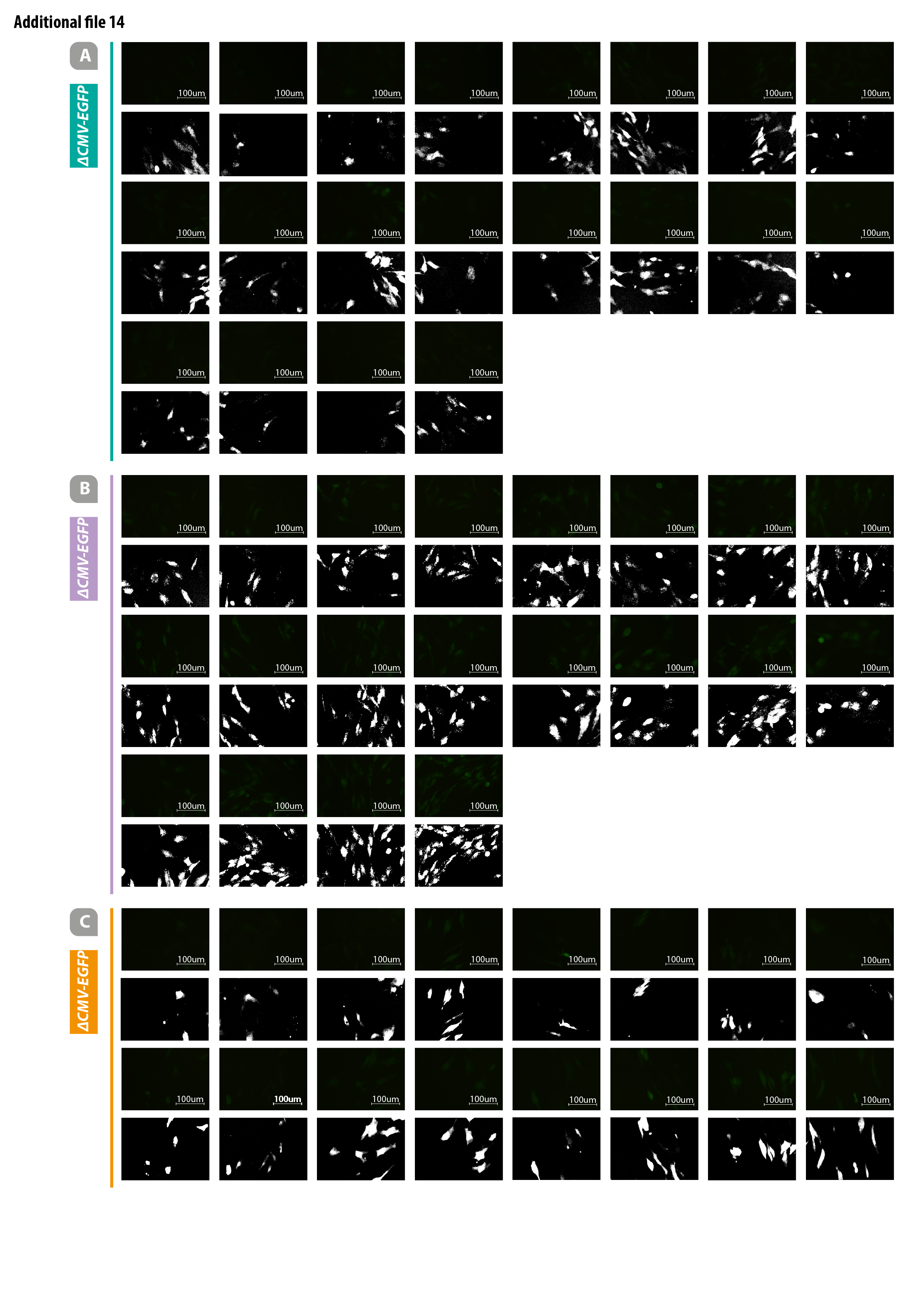

Supplement: Supplementary file 14 — Additional file 14. Images of heterogenous cell pools harboring ΔCMV-EGFP for analyzing by ImageJ software. [file 12575_2023_210_MOESM14_ESM.zip › (additional file 14) Proof version-01_ESM.tif]

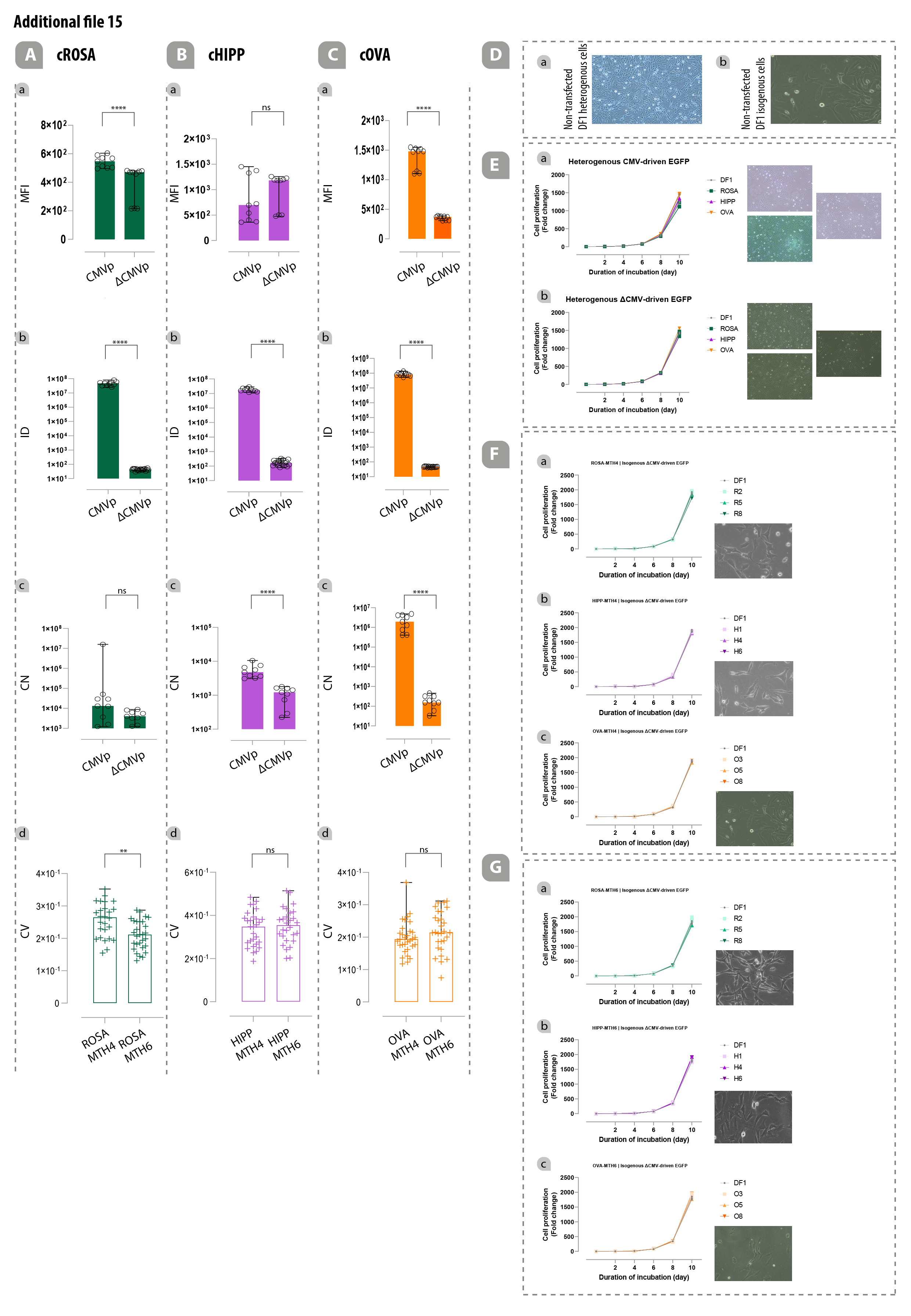

Supplement: Supplementary file 15 — Additional file 15. Comparison of EGFP expression levels in heterogenous cell pools and isogenous cell clones harboring CMV-driven EGFP or ∆CMV-driven EGFP and doubling time and morphology of targeted cells. [file 12575_2023_210_MOESM15_ESM.zip › (additional file 15) Proof version-01_ESM.tif]

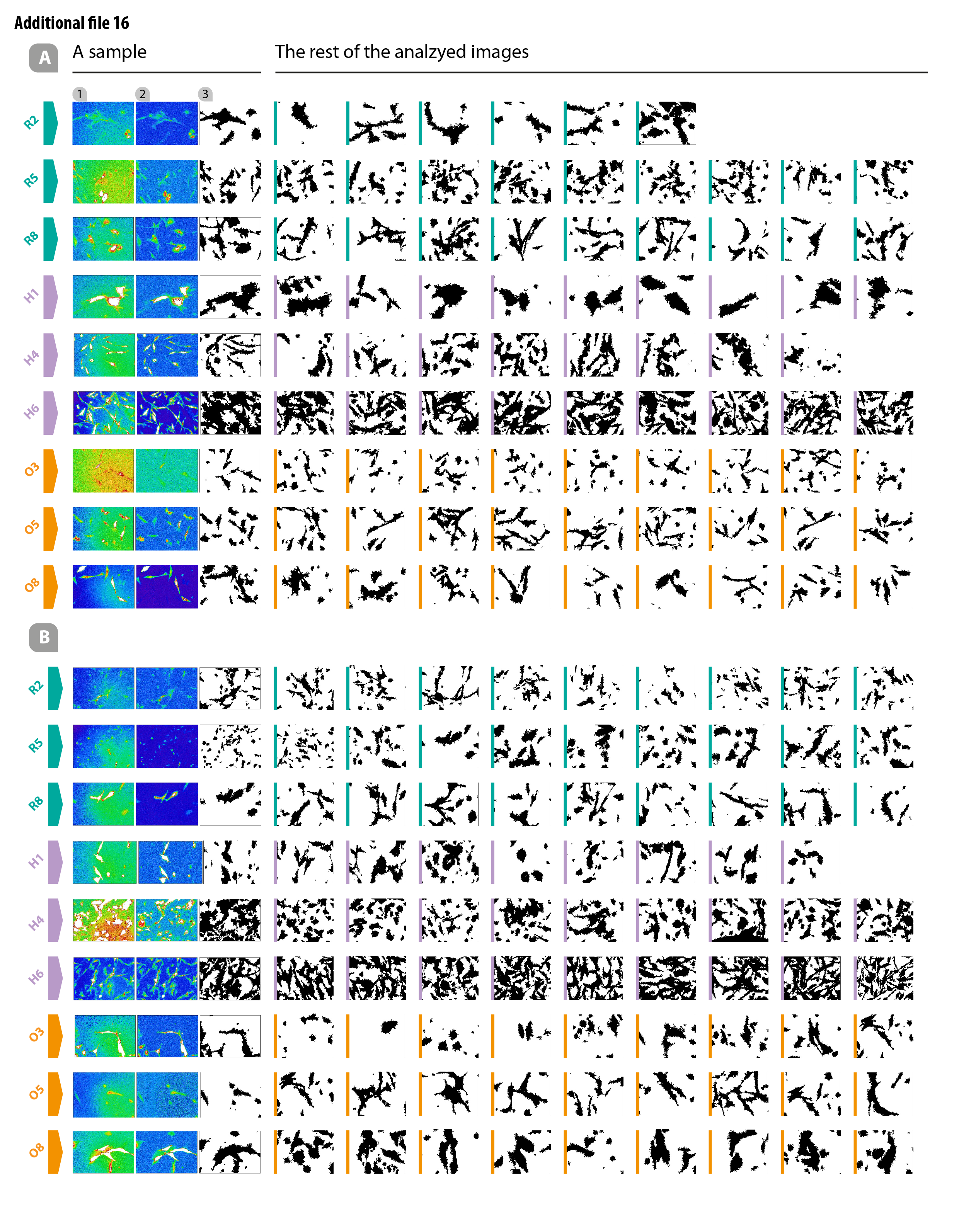

Supplement: Supplementary file 16 — Additional file 16. Images of isogenous cell clones harboring the DsRed2-ΔCMV-EGFP transgene. [file 12575_2023_210_MOESM16_ESM.zip › (additional file 16) Proof version-01_ESM.tif]

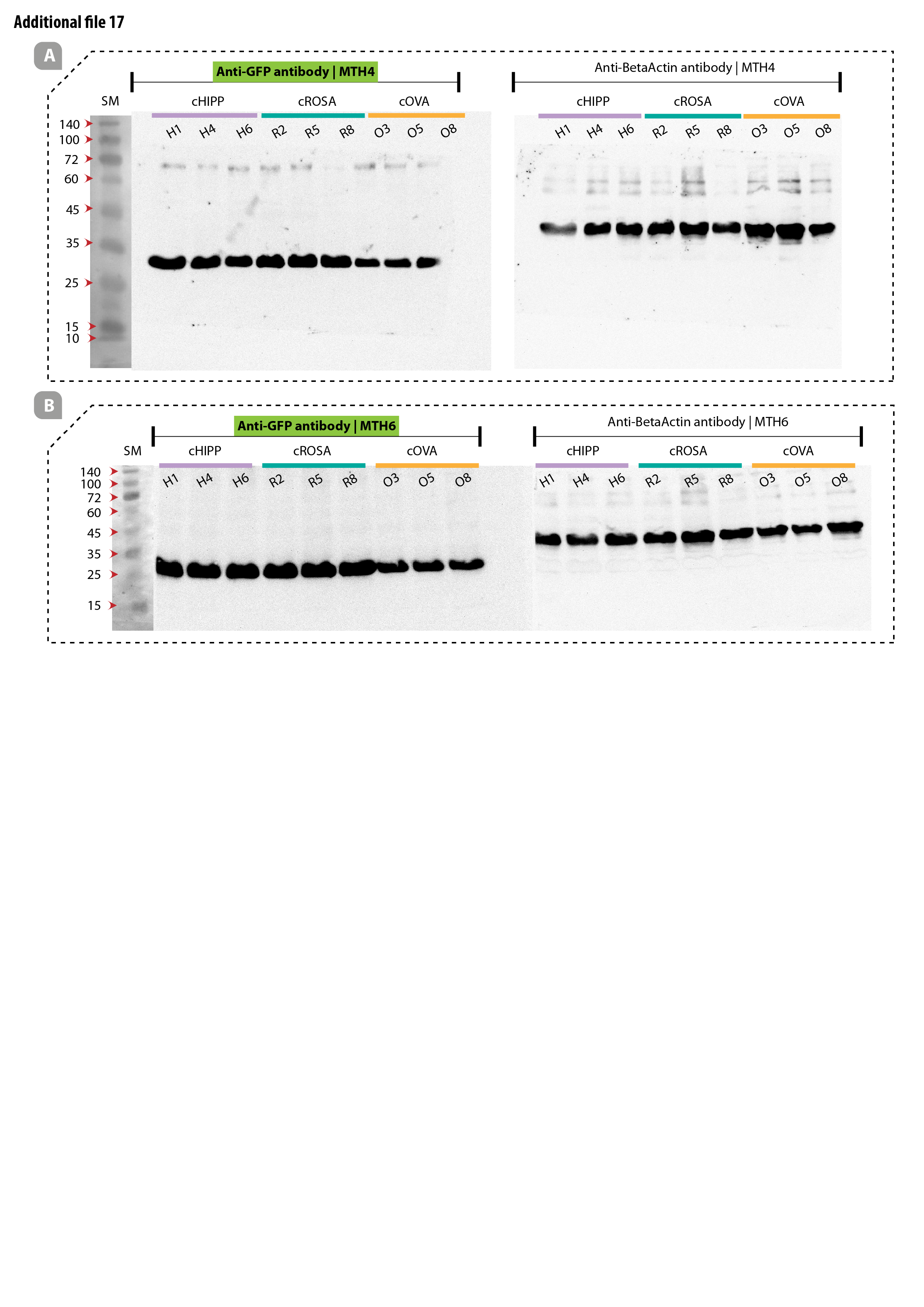

Supplement: Supplementary file 17 — Additional file 17. Western blot analysis for evaluating the expression of the EGFP protein. [file 12575_2023_210_MOESM17_ESM.zip › (additional file 17) Proof version-01_ESM.tif]
