# Supplementary material for: Integrating Omics and CRISPR Technology for Identification and Verification of Genomic Safe Harbor Loci in the Chicken Genome
Source: Biol Proced Online. 2023 Jun 24;25:18. doi: 10.1186/s12575-023-00210-5 (PMC10290409; doi:10.1186/s12575-023-00210-5)
Supplement: Supplementary file 7 — Additional file 7. Different cell lines generated and used in this study. [file 12575_2023_210_MOESM7_ESM.zip › (additional file 7) Legend - Proof version_ESM.docx]

**Additional file 7.** Different cell lines generated and used in this study

A) Schematic depiction of the experimental procedure timeline. B) At the end of month 2 (MTH2), three kinds of DF1 heterogeneous cells were generated that contained the DsRed2-CMV-EGFP transgene integrated site-specifically into the cROSA, cHIPP, and cOVA loci. C) At the end of month 2 (MTH2), three kinds of DF1 heterogeneous cells were generated that contained the DsRed2-ΔCMV-EGFP transgene integrated site-specifically into the cROSA, cHIPP, and cOVA loci. D) At the end of month 4 (MTH4), a population of DF1 isogenous cell clones was clonally expanded from the MTH2 heterogeneous cell pools harboring the DsRed2-ΔCMV-EGFP transgene. E) At the end of month 6 (MTH6), a population of DF1 isogenous cell clones was further expanded from the MTH4 isogenous cell clones harboring the DsRed2-ΔCMV-EGFP transgene. The schematic territories of the cROSA, cHIPP, and cOVA loci in their TADs have been illustrated by cyan, purple, and orange colors. Red diamonds are the approximate integration sites of the transgene. (d: day; MTH: month)
