# Supplementary material for: Integrating Omics and CRISPR Technology for Identification and Verification of Genomic Safe Harbor Loci in the Chicken Genome
Source: Biol Proced Online. 2023 Jun 24;25:18. doi: 10.1186/s12575-023-00210-5 (PMC10290409; doi:10.1186/s12575-023-00210-5)
Supplement: Supplementary file 8 — Additional file 8. Isolating isogenous cell clones, verification of correctly-targeted clones, and confirmation of mono-allelic and single copy knocked-in transgenes in GSH and non-GSH loci. [file 12575_2023_210_MOESM8_ESM.zip › (additional file 8) Legend - Proof version_ESM.docx]

**Additional file 8.** Isolating isogenous cell clones, verification of correctly-targeted clones, and confirmation of mono-allelic and single copy knocked-in transgenes in GSH and non-GSH loci

A) Isolating single-cell clones from correctly-targeted heterogenous cell pools (cROSA, cHIPP, and cOVA) by limit diluting method and picking up the isogenous cell clones and culturing and expanding in 12 well plates. B) Screening the isolated green clones for finding bi- or mono-allelic knock-ins. C) Screening mono-allelic knocked-in clones for true knock-in by genomic PCR on the junctions. D) Confirmation of the mono-allelic targeted clones by restriction enzyme digestion. E) Screening the mono-allelic targeted clones for finding single-copy knock-in.
