# Supplementary material for: Integrating Omics and CRISPR Technology for Identification and Verification of Genomic Safe Harbor Loci in the Chicken Genome
Source: Biol Proced Online. 2023 Jun 24;25:18. doi: 10.1186/s12575-023-00210-5 (PMC10290409; doi:10.1186/s12575-023-00210-5)
Supplement: Supplementary file 9 — Additional file 9. Verification of CRISPR-mediated knock-ins in the chicken GSH loci and non-GSH locus. [file 12575_2023_210_MOESM9_ESM.zip › (additional file 9) Legend - Proof version_ESM.docx]

**Additional file 9.** Verification of CRISPR-mediated knock-ins in the chicken GSH loci and non-GSH locus.

Heterogeneous cell pools harboring the DsRed2-CMV-EGFP or DsRed2-ΔCMV-EGFP transgene were subjected to 5’/3’ junction PCR amplification using genome-specific (GS) and vector-specific (VS) primers and restriction enzyme digestion of the amplicons. For the 5’ junction PCR, targeted integration of VR, VH, VO, ΔVR, ΔVH, and ΔVO was verified by amplification of 1086-bp, 1181-bp, and 1188-bp amplicons, respectively from the 5’-side of each locus (A-a and A-b for the cROSA locus; B-a and B-b for the cHIPP locus; C-a and C-b for the cOVA locus). For the 3’ junction PCR, the 678-bp, 753-bp, and 1098-bp amplicons correspond to the 3’-side of cROSA, cHIPP, and cOVA, respectively (A-d and A-e for the cROSA-like locus; B-d and B-e for the cHIPP-like locus; C-d and C-e for the cOVA locus). A-c) Restriction enzyme digestion by BsrGI and PvuII confirmed the accuracy of amplicons from 5’ and 3’ junctions of cROSA locus, respectively. B-c) Restriction enzyme digestion by ScaI and SacI confirmed the accuracy of amplicons from 5’ and 3’ junctions of cHIPP locus, respectively. C-c) Restriction enzyme digestion by BglII and NheI confirmed the accuracy of amplicons from 5’ and 3’ junctions of cOVA locus, respectively.

SM: size marker; EXP.: experiment; GS: genome-specific primer; VS: vector-specific primer; VR: ROSA-targeting vector with CMV promoter; ΔVR: ROSA-targeting vector with ΔCMV promoter; VH: HIPP-targeting vector with CMV promoter; ΔVH: HIPP-targeting vector with ΔCMV promoter; VO: OVA-targeting vector with CMV promoter; ΔVO: OVA-targeting vector with ΔCMV promoter; -gRNA: a px459 vector without sgRNA. Amplicons marked by * or ** correspond to restriction enzyme digestion marked by * or **. Red arrows point to the correct amplicons in each experimental group.
