# Supplementary material for: Integrating Omics and CRISPR Technology for Identification and Verification of Genomic Safe Harbor Loci in the Chicken Genome
Source: Biol Proced Online. 2023 Jun 24;25:18. doi: 10.1186/s12575-023-00210-5 (PMC10290409; doi:10.1186/s12575-023-00210-5)
Supplement: Supplementary file 10 — Additional file 10. Sanger sequencing analysis of 5’- and 3’-flanking junctions of correctly-targeted GSH loci and non-GSH locus. [file 12575_2023_210_MOESM10_ESM.zip › (additional file 10) Legend - Proof version_ESM.docx]

**Additional File 10.** Sanger sequencing analysis of 5’- and 3’-flanking junctions of correctly-targeted GSH loci and non-GSH locus

The correctly-targeted chicken GSH loci and non-GSH locus were verified using Sanger sequencing analysis of 5’- and 3’-flanking junctions. A part of the original sequence, 5’- or 3’-junctions, and left or right homology arms (LHA or RHA, respectively) are shown for each locus. Chromatograms for verification of 5’- and 3’-flanking junctions of correctly targeted heterogenous cell pools harboring either CMV-EGFP or ΔCMV-EGFP in cROSA (A-a and A-b), cHIPP (B-a and B-b), and cOVA (C-a and C-b) loci were shown. Also, chromatograms for verification of 5’- and 3’-flanking junctions of correctly targeted isogenous cell clones (R2, R5, and R8 clones; H1, H4, and H6 clones; O3, O5, and O8 clones) harboring ΔCMV-EGFP in cROSA (A-c), cHIPP (B-c), and cOVA (C-c) loci were shown.
