# Supplementary material for: Integrating Omics and CRISPR Technology for Identification and Verification of Genomic Safe Harbor Loci in the Chicken Genome
Source: Biol Proced Online. 2023 Jun 24;25:18. doi: 10.1186/s12575-023-00210-5 (PMC10290409; doi:10.1186/s12575-023-00210-5)
Supplement: Supplementary file 11 — Additional file 11. GNUastro command lines used for analysis of images. [file 12575_2023_210_MOESM11_ESM.docx]

**Additional File 11.** GNUastro command lines used for analysis of images

GNUastro software was downloaded using the following command in the Ubuntu software (Linux-based software):

- wget akhlaghi.org/gnuastro-latest.tar.lz

Converting all images to FITS format using following command:

- astconvertt $(INDIR)/$*.tif -h0 -o$$input

separating the green channel from the others (red and blue) using following command:

- mecub $(INDIR)/$*.fits

Removing background gradient and detecting over the green channel using following command:

- Astnoisechisel $$input -h2 --tilesize=10,10 --meanmedqdiff=0.01 --outliersigma=50 --qthresh=0.6
  --detgrowquant=0.99 --output=$$nc

Analyzing FITS images and converting them into JPEG format in the green channel using following command:

- Astconvertt blank $$nc blank -hINPUT-NO-SKY –fluxhigh=10 -o$(BDIR)/$*/in.jpg

Calculating mean fluorescence intensity (MFI) using following command:

- Astarithmetic $(BDIR)/$*/nc.fits -hINPUT-NO-SKY set-value $(BDIR)/$*/nc.fits -hDETECTIONS set-det value det 0 eq nan where set-use use meanvalue --quiet > o.txt
