# Supplementary material for: Integrating Omics and CRISPR Technology for Identification and Verification of Genomic Safe Harbor Loci in the Chicken Genome
Source: Biol Proced Online. 2023 Jun 24;25:18. doi: 10.1186/s12575-023-00210-5 (PMC10290409; doi:10.1186/s12575-023-00210-5)
Supplement: Supplementary file 12 — Additional file 12. The coordinates of cROSA, cHIPP, and cOVA loci as well as flanking genes have been visualized by JUICEBOX online software (the coordinate system of the map corresponds to the genome version GalGal5). [file 12575_2023_210_MOESM12_ESM.zip › (additional file 12) Legend - Proof version_ESM.docx]

**Additional file 12.** The coordinates of cROSA, cHIPP, and cOVA loci as well as flanking genes have been visualized by JUICEBOX online software (the coordinate system of the map corresponds to the genome version GalGal5).

a) cROSA locus has been located between 19280000 and 19520000 coordinates in chromosome 12. b) cHIPP locus has been located between 9040000 and 9320000 coordinates in chromosome 15. c) cOVA locus has been located between 67840000 and 68040000 coordinates in chromosome 2. (In general, block circles show TAD boundaries; block arrows show integration site of transgene; X- and Y-axis show coordinates of the map of the genome).
