# Supplementary material for: Integrating Omics and CRISPR Technology for Identification and Verification of Genomic Safe Harbor Loci in the Chicken Genome
Source: Biol Proced Online. 2023 Jun 24;25:18. doi: 10.1186/s12575-023-00210-5 (PMC10290409; doi:10.1186/s12575-023-00210-5)
Supplement: Supplementary file 13 — Additional file 13. Images of heterogenous cell pools harboring CMV-EGFP for analyzing by ImageJ software. [file 12575_2023_210_MOESM13_ESM.zip › (additional file 13) Legend - Proof version_ESM.docx]

**Additional file 13.** Images of heterogenous cell pools harboring CMV-EGFP for analyzing by ImageJ software

All images were captured in 10X magnification, 100µs exposure, and 1X analog gain (scale bar 100µm). Original images captured by fluorescence microscope and analyzed images by ImageJ software (dark background images) have been shown.
