# Supplementary material for: Integrating Omics and CRISPR Technology for Identification and Verification of Genomic Safe Harbor Loci in the Chicken Genome
Source: Biol Proced Online. 2023 Jun 24;25:18. doi: 10.1186/s12575-023-00210-5 (PMC10290409; doi:10.1186/s12575-023-00210-5)
Supplement: Supplementary file 15 — Additional file 15. Comparison of EGFP expression levels in heterogenous cell pools and isogenous cell clones harboring CMV-driven EGFP or ∆CMV-driven EGFP and doubling time and morphology of targeted cells. [file 12575_2023_210_MOESM15_ESM.zip › (additional file 15) Legend - Proof version_ESM.docx]

**Additional file 15.** Comparison of EGFP expression levels in heterogenous cell pools and isogenous cell clones harboring CMV-driven EGFP or $\Delta$CMV-driven EGFP and doubling time and morphology of targeted cells.

Impact of promoter shortening on transcription status and expression level of EGFP compared to full-length CMV. Mean fluorescence Intensity index (A-a, B-a, and C-a), Integrated Density index (A-b, B-b, and C-b), qPCR results (A-c, B-c, C-c), and the Coefficient of Variation of Integrated Density index (A-d, B-d, C-d) of heterogenous cell pools harboring CMV-EGFP have been compared with these parameters in the heterogenous cell pools harboring $\Delta$CMV-EGFP in each locus. The morphology and doubling time of targeted cells were compared with wild-type controls. D-a, D-b) Heterogenous and isogenous wild-type controls. E-a) Comparison of doubling time and morphology among heterogenous cell pools harboring CMV-EGFP with wild-type controls. E-b) Comparison of doubling time and morphology among heterogenous cell pools harboring $\boldsymbol{\Delta}$CMV-EGFP with wild-type controls. F-a, F-b, F-c) Comparison of doubling time and morphology among isogenous cell clones harboring $\boldsymbol{\Delta}$CMV-EGFP with wild-type controls at the end of month 4. G-a, G-b, G-c) Comparison of doubling time and morphology among isogenous cell clones harboring $\boldsymbol{\Delta}$CMV-EGFP with wild-type controls at the end of month 6.
