# Supplementary material for: Integrating Omics and CRISPR Technology for Identification and Verification of Genomic Safe Harbor Loci in the Chicken Genome
Source: Biol Proced Online. 2023 Jun 24;25:18. doi: 10.1186/s12575-023-00210-5 (PMC10290409; doi:10.1186/s12575-023-00210-5)
Supplement: Supplementary file 16 — Additional file 16. Images of isogenous cell clones harboring the DsRed2-ΔCMV-EGFP transgene. [file 12575_2023_210_MOESM16_ESM.zip › (additional file 16) Legend - Proof version_ESM.docx]

**Additional file 16.** Images of isogenous cell clones harboring the DsRed2-ΔCMV-EGFP transgene

All images were captured at 20X magnification, 1s exposure, and 1X analog gain (scale bar: 100µm). Images for all clones (R2, R5, R8, H1, H4, H6, O3, O5, and O8) at the end of MTH4 and MTH 6 were analyzed by the GNUastro software (dark cells and white background). Columns 1 to 3 show an example processing of pictures from the original to polished, and polished to the final images (which were used in the analysis).
