# Supplementary material for: Integrating Omics and CRISPR Technology for Identification and Verification of Genomic Safe Harbor Loci in the Chicken Genome
Source: Biol Proced Online. 2023 Jun 24;25:18. doi: 10.1186/s12575-023-00210-5 (PMC10290409; doi:10.1186/s12575-023-00210-5)
Supplement: Supplementary file 17 — Additional file 17. Western blot analysis for evaluating the expression of the EGFP protein. [file 12575_2023_210_MOESM17_ESM.zip › (additional file 17) Legend - Proof version_ESM.docx]

**Additional file 17.** Western blot analysis for evaluating the expression of the EGFP protein

The expression level of EGFP protein originating from the targeted loci in parallel with the level of beta-actin protein was determined at the end of month 4 (A) and month 6 (B).
